# Mapping research landscapes: a bibliometric and visual analysis of ketogenic diet interventions in liver health (2013–2024)

**DOI:** 10.3389/fnut.2025.1652271

**Published:** 2025-12-23

**Authors:** Yinglian Wang, Qingliu Lu, Hailin Ma, Jing’an Huang, Bo Zhang, Yunyun Qin, Zhongwen Feng, Xuefeng Jin

**Affiliations:** 1 Department of Nursing, Guangxi Academy of Medical Sciences and the People’s Hospital of Guangxi Zhuang Autonomous Region, Nanning, China; 2 Department of Pharmacy, Guangxi Academy of Medical Sciences and the People’s Hospital of Guangxi Zhuang Autonomous Region, Nanning, China; 3 Scientific Research Center, Guilin Medical University, Guilin, China

**Keywords:** bibliometric analysis, ketogenic diet, non-alcoholic fatty liver disease, alcoholic hepatitis, liver cirrhosis, hepatocellular carcinoma

## Abstract

**Objective:**

Bibliometric and visual analysis in the field of ketogenic diet (KD) on Liver Health from 2013 to 2024.

**Methods:**

We retrieved the articles published between 2013 and 2024 from the Web of Science database and the Scopus database, and conducted the analysis using R software and VOSviewer software.

**Results:**

The number of publications in this field shows an increasing trend year by year. The United States leads in the number of published articles, followed closely by China, Italy, Japan, and Canada. Notably, the United States has also excelled in international collaboration, with institutions like Sapienza University of Rome and the University of California, San Francisco, actively engaging with other global institutions. *Nutrients* has the highest publication frequency, while *Cell Metabolism* leads in citations. Key researchers such as Crawford PA and Watanabe M have emerged, with prominent keywords including obesity, Metabolic Associated Steatosis Liver Disease, NAFLD, beta-hydroxybutyrate, and low carbohydrate diet, indicating the central themes and trends in KD related Liver Health research.

**Conclusion:**

KD, a novel dietary therapy designed to induce physiological ketosis, is anticipated to achieve significant advances in liver health. Global interest in this approach is increasing, underscoring its potential as an emerging therapeutic trend. This study offers a thorough analysis of the current research landscape and key hotspots related to the KD in liver health, providing valuable insights for future investigations.

## 1 Introduction

The liver is a vital organ responsible for essential functions in digestion, metabolism, nutrient storage and release, and regulation of peroxide free radical levels. Its health is crucial for maintaining normal physiological processes, making liver diseases a major concern (1). Recent research indicates that modern dietary habits and lifestyles have contributed to the rising prevalence of liver conditions such as NAFLD (2), alcoholic hepatitis (ALD) (3), liver cirrhosis, and hepatocellular carcinoma (HCC) (4, 5). These liver diseases significantly impact individuals’ quality of life, pose serious health risks, and reduce longevity. NAFLD, the most common liver disorder, is closely associated with obesity, insulin resistance, and metabolic syndrome (6). Although early stages of these diseases can be asymptomatic, they often progress to more severe liver damage, including cirrhosis and cancer, without timely intervention (7).

Management of NAFLD generally focuses on lifestyle modifications, including dietary interventions that emphasize low-calorie, low-fat, high-fiber diets, and reduced sugar intake (8). Approaches such as the Mediterranean diet have been advocated for their beneficial effects on liver health (8). Pharmacological treatments, including insulin sensitizers like metformin (9), vitamin E (10), and emerging anti-obesity agents like glucagon-like peptide-1 (GLP-1) agonists and sodium-glucose cotransporter-2 (SGLT-2) inhibitors (11, 12), can also be used to address metabolic disturbances. In addition, aerobic exercise and weight management play a crucial role in improving liver function (13). However, long-term adherence to these lifestyle changes remains challenging for many patients, and liver health improvements often take time to manifest. For ALD, the primary treatment is complete abstinence from alcohol, which helps reduce oxidative stress and inflammation, thereby improving liver function (14). Pharmacological interventions, including corticosteroids and antioxidants such as N-acetylcysteine, may complement this approach, but not all patients respond favorably, and potential side effects must be considered (15).

In early-stage cirrhosis, the treatment is primarily supportive, focusing on symptom management and monitoring of liver function (16). Diuretics for ascites, beta-blockers to reduce the risk of esophageal variceal bleeding, and antiviral therapies for hepatitis B and C are commonly employed (17–19). However, the progression of cirrhosis remains largely irreversible, with current treatments mainly aiming at disease management rather than a cure (20). HCC, a leading cause of liver-related deaths, remains a major therapeutic challenge (21). Treatment options include targeted therapy and immunotherapy, with first-line drugs such as sorafenib and lenvatinib demonstrating inhibitory effects on tumor growth (22). However, these treatments can lead to drug resistance over time, limiting their effectiveness. Immunotherapy, which aims to harness the body’s immune system to fight cancer, faces challenges related to immune escape mechanisms in tumor cells (23). As a result, the development of new therapeutic strategies is essential to improve liver disease outcomes.

The ketogenic diet (KD), characterized by high-fat, moderate-protein, and low-carbohydrate intake, induces a metabolic state known as ketosis (24). Ketosis has gained attention for its potential benefits, particularly in the context of liver health. The KD reduces carbohydrate intake, lowering blood sugar levels and improving insulin sensitivity, which in turn can reduce insulin secretion (25). This reduction in insulin has a positive effect on liver fat metabolism, helping to prevent fat accumulation and manage fatty liver disease (26). Furthermore, research suggests that the KD influences liver lipid metabolism and reduces oxidative stress (27). Ketones produced during ketosis may protect liver cells by modulating insulin signaling pathways and enhancing mitochondrial oxygen metabolism, potentially alleviating oxidative damage in early-stage cirrhosis (28). This metabolic shift may also help prevent the progression from fatty liver to liver fibrosis. Additionally, the KD has shown promise in cancer treatment by inhibiting the Warburg effect in tumor cells (29). It reduces oxidative stress in normal cells while intensifying it in tumor cells, thereby promoting their apoptosis and autophagy (30). Furthermore, microRNAs (miRNAs) can serve as biomarkers of specific liver damage and have a significant impact on liver health (31). The ketogenic diet can interfere with the function of specific miRNAs and reshape the body (32). However, while these results are promising, current research lacks a comprehensive analysis of the KD’s full impact on liver health, making it difficult to draw clear conclusions about its long-term effectiveness and optimal application in clinical settings.

Bibliometric analysis is a powerful tool for visualizing trends and relationships in scientific research by quantitatively assessing large bodies of published work (33). This method can help identify key authors, institutions, countries, and collaborative networks within a particular field. By analyzing the literature on the KD’s role in liver health from 2013 to 2024, the present study aims to fill the gap in the current literature by providing a comprehensive overview of research trends, hotspots, and emerging areas of interest. This analysis will allow researchers to better understand the progression of knowledge in this field and identify key knowledge gaps. Using tools such as VOSviewer, R software, and CiteSpace, this study will visually map the evolution of research on the KD and liver health, helping to pinpoint critical knowledge advancements and areas that require further investigation. The findings will provide valuable insights into the future direction of research, fostering interdisciplinary collaboration and helping guide new therapeutic strategies in the field of liver health and dietary interventions.

## 2 Materials and methods

### 2.1 Literature sources and retrieval strategies

On July 21, 2024, retrievals were conducted, respectively, in the Web of Science Core Collection (WoSCC) and Scopus databases. The search strategies of WoSCC and Scopus database can be found in Supplementary material 1. The inclusion and exclusion criteria of the literature in this study were strictly set according to the search formula. The retrieved literatures in WoSCC were saved in plain text format and exported as complete records, including cited references. The retrieved literatures in Scopus were saved in csv format and exported as complete records, including cited references. With the help of Excel software, we achieve the goal of eliminating duplicate literature by excluding the same DOI numbers. Considering that merging data generated from different databases would lead to a large amount of data loss. For instance, citation data will be impossible to analyze. Therefore, to ensure the integrity of the data, we will analyze different databases separately. After obtaining the analysis results from different databases, we conduct a comprehensive analysis to identify the research hotspots in this field.

### 2.2 Date analysis

Given the discrepancies in data formats between the WoSCC and Scopus databases, combining them would lead to data loss. As such, we will conduct separate analyses on the data from each database to achieve more reliable outcomes. Moreover, it should be noted that, considering the high-quality nature of the literature included in WoSCC, our primary focus for subsequent analysis will be on the data from WoSCC. The analysis results of the Scopus database, including annual publication trends and keyword clustering, will be provided in the Supplementary materials.

To analyze the annual publications, Origin 2018 was used. Additionally, the bibliometrix package of R software (version 4.3.1), VOSviewer (version 1.6.18), and CiteSpace (version 6.3.1.0) were employed to visually analyze data and draw scientific knowledge maps.

The VOSviewer software was employed to create visualizations of national collaboration networks, source co-citation analysis, and keyword co-occurrence. The specific parameters utilized in the VOSviewer analysis can be located in Supplementary material 2. Additionally, the Impact Factors (IF) of the journals were obtained from the 2023 Journal Citation Reports (JCR).

## 3 Results

### 3.1 General landscapes of included documents on KD on liver health

**Figure 1:**
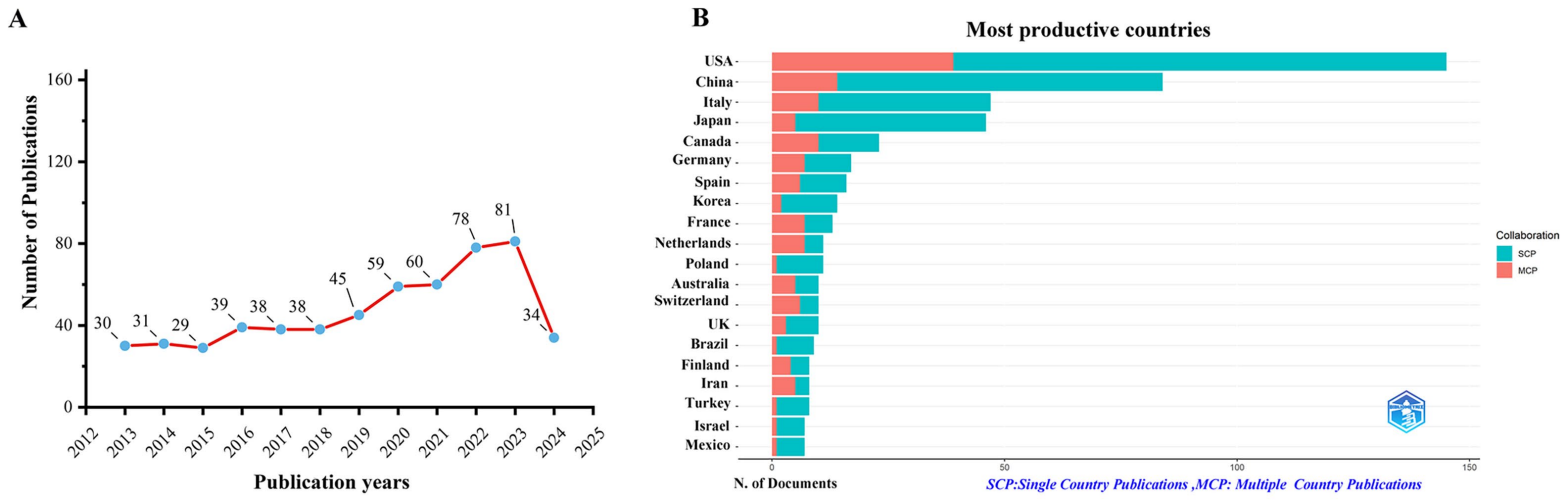
Trends in annual publication outputs in the field of KD in liver health from 2013 to 2024. **(A)** Trends of annual publication outputs. **(B)** Showcases the distribution of countries and collaborative efforts among corresponding authors.

The total number of unique documents retrieved from WoSCC is 561. In Figure 1A, from 2013 to 2024, the number of publications in this field shows a general upward trend. Notably, between 2019 and 2022, the rate of increase was particularly steep. The highest number of publications occurred in 2023, with a total of 81. Additionally, as of July 21, 2024, there have already been 34 publications, further contributing to the growing body of literature in this area. A total of 1,402 unique records were collected from Scopus database, with duplicates removed. The growth trend of publications is consistent with that of WoSCC (Supplementary Figure S1). This suggests a rising interest in the topic of KD for liver health in recent years.

**Figure 2:**
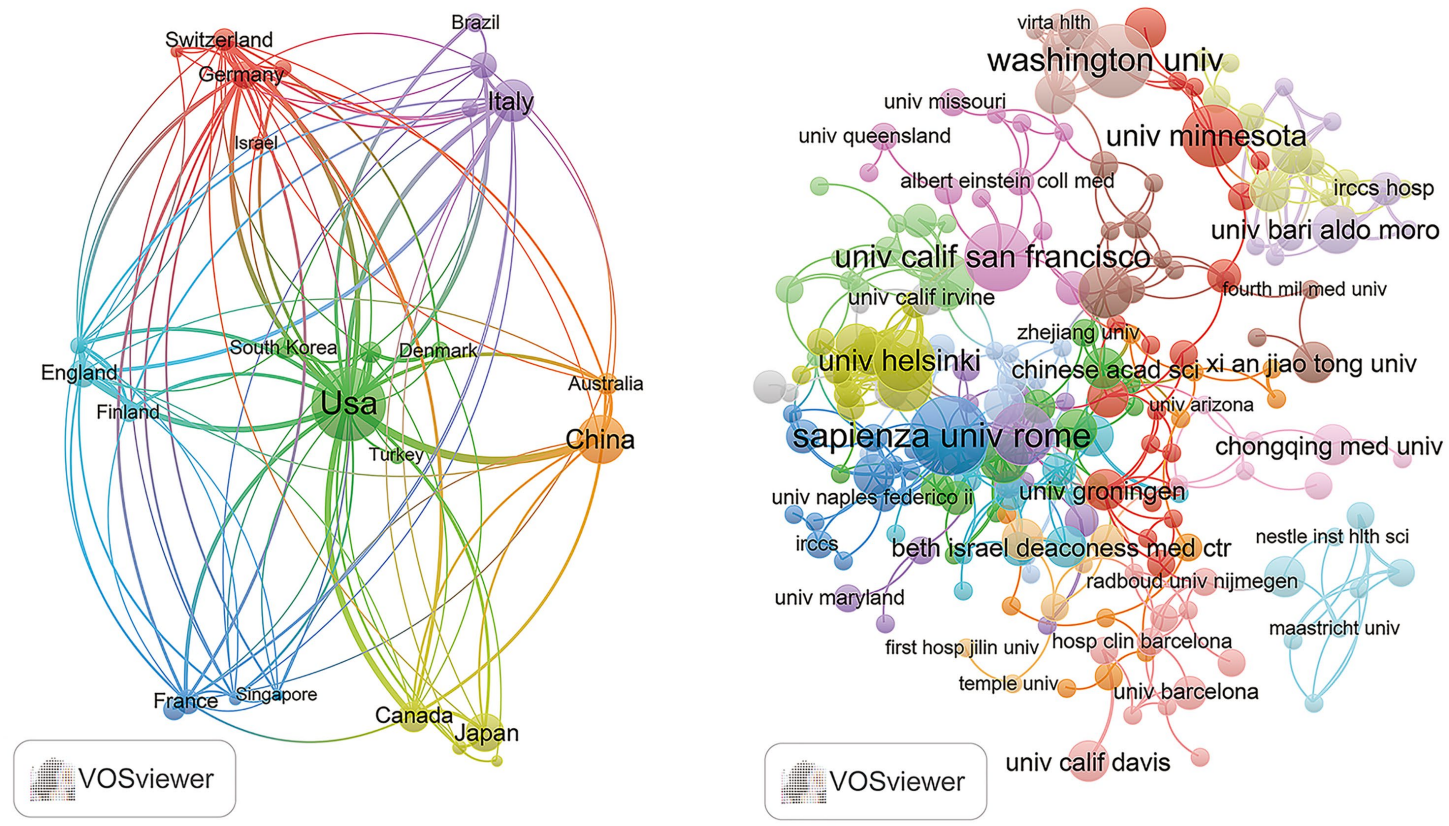
The map of countries and institutions in the field of KD in liver health from 2013 to 2024.

The top five countries in this field are the United States with (*n* = 145), China (*n* = 84), Italy (*n* = 47), Japan (*n* = 46), and Canada (*n* = 23). Among them, the United States has published the most papers, indicating its leading position and significant contributions in this field. Furthermore, among the top 10 countries in this field, Norway (64.00%) and France (54.00%) have the highest proportion of multiple country publications (MCPs). This suggests a strong emphasis on international collaboration and academic exchange within these two countries. Conversely, Japan (11.00%) and South Korea (14.00%), indicating a greater focus on local originality in their research endeavors (Figure 1B; Table 1). Furthermore, Figure 2A shows that the United States is the cooperation center in this field. It indicates that the United States plays an important role in promoting the development of this field. Of the top 10 published research institutions, five are from US, two from France, and one each from China and Italy. The Sapienza University Rome, an Italian institution, published the most papers (*n* = 11), following behind are two American institutions Washington University (*n* = 10), and California San Franciso University (*n* = 9). This indicates that the field remains in its nascent stages (Figure 2B; Table 2).

**Table 1:** Most relevant countries by corresponding authors of KD on liver health.

Country	Articles	SCP	MCP	Freq (%)	MCP_ratio (%)
USA	145	106	39	26%	27%
China	84	70	14	15%	17%
Italy	47	37	10	8%	21%
Japan	46	41	5	8%	11%
Canada	23	13	10	4%	44%
Germany	17	10	7	3%	41%
Spain	16	10	6	3%	38%
Korea	14	12	2	3%	14%
France	13	6	7	2%	54%
Netherlands	11	4	7	2%	64%

Multiple-country publication (MCP); Single-country publication (SCP).

**Table 2:** Top 10 most relevant affiliations of KD on liver health.

Rank	Affiliation	Country	Articles (*n*)
1	Sapienza University Rome	Italy	11
2	Washington University	USA	10
3	California San Franciso University	USA	9
4	Harvard Medical School	USA	8
5	Helsinki University	Finland	8
6	Minnesota University	USA	8
7	Calgary University	Canada	7
8	Inserm	France	7
9	Oxford University	United Kingdom	7
10	Shanghai Jiao Tong University	China	7

### 3.2 Journals and co-cited journals

The most cited articles and journals in this field were analyzed using the bibliometrix and ggplot2 packages in R software (4.22.17675.0).

**Figure 3:**
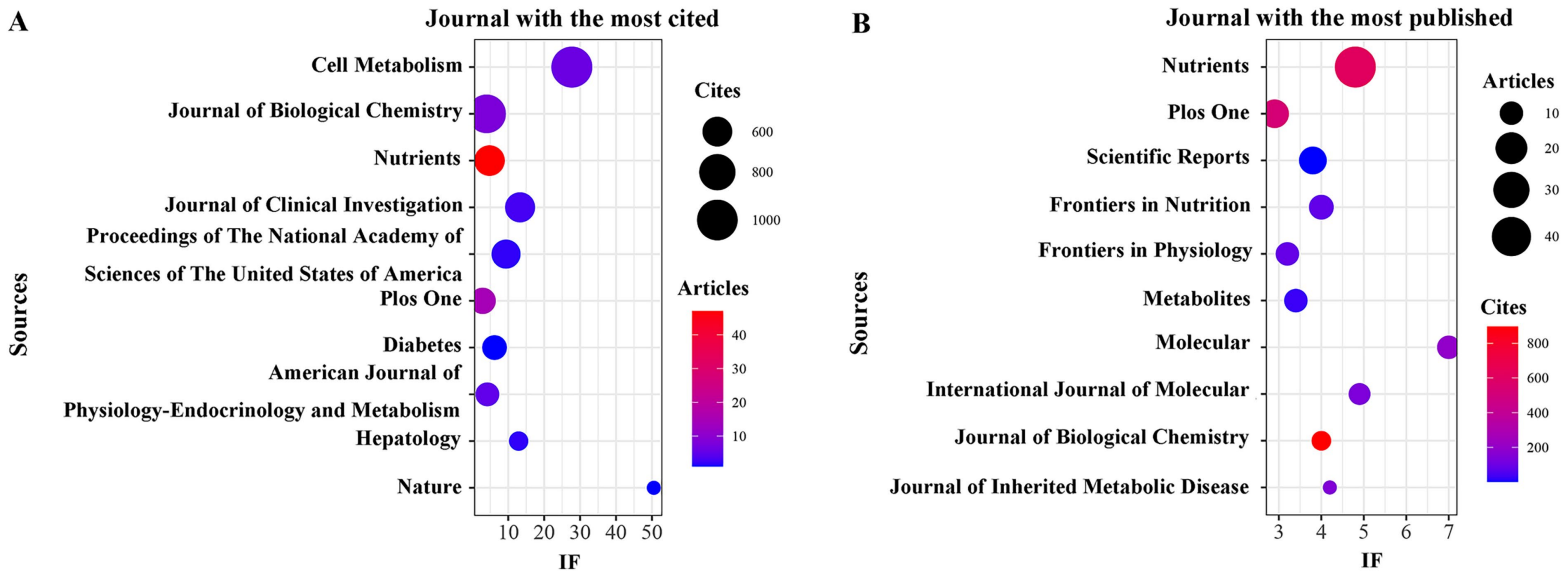
The journal with the largest number of articles published and the journal with the largest number of citations. **(A)** The journal with the highest count of published documents. **(B)** The journals with the highest count of citations.

**Figure 4:**
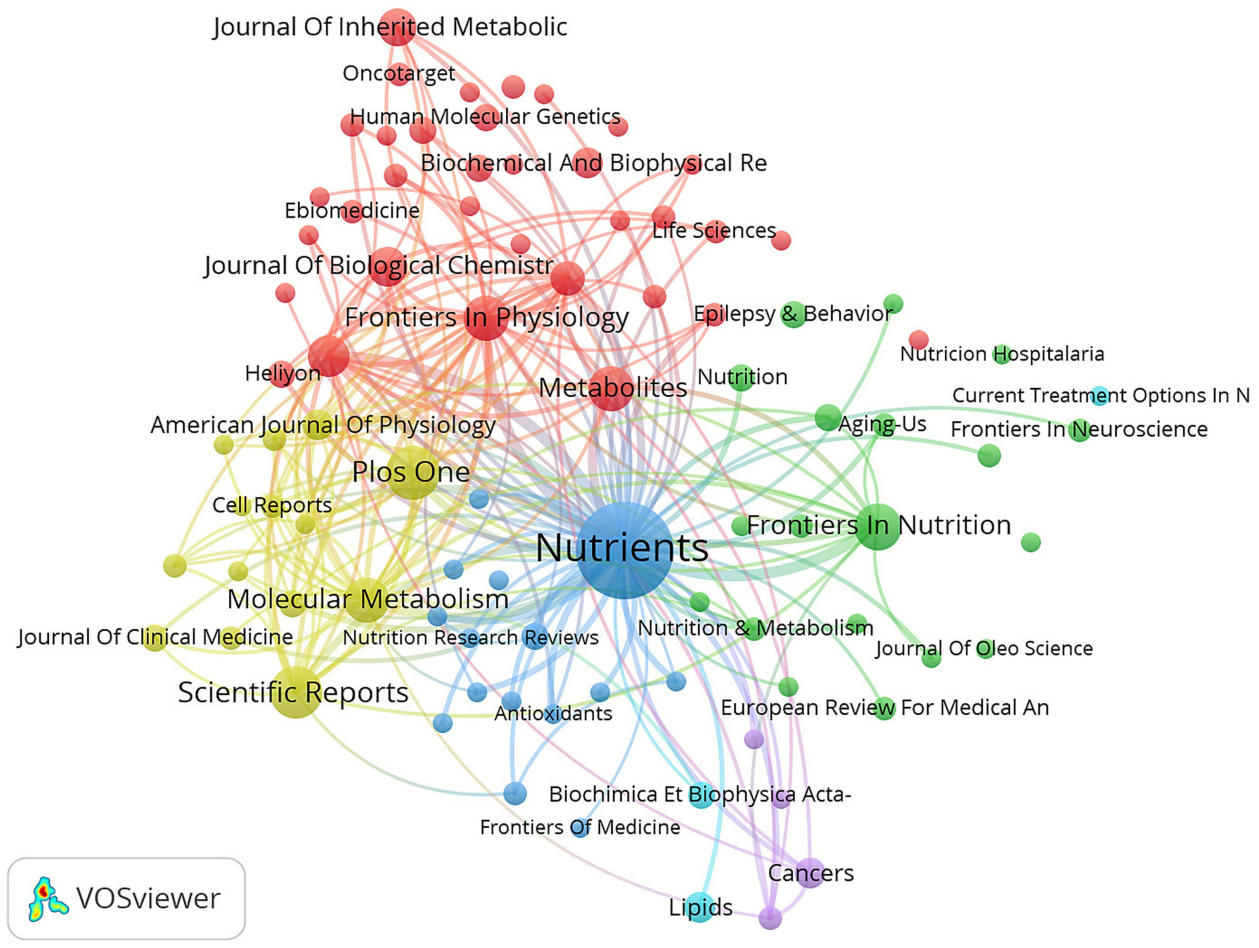
Co-cited journals involved in the field of the field of KD in liver health.

In addition, VOSviewer (version 1.6.18.0) was used for co-citation journal analysis. The results showed that a total of 561 papers were published in 292 academic journals (Table 3). Figure 3A shows that the journal with the highest citation frequency is *Cell Metabolism* (*n* = 1,262, IF = 27.7), followed by *Nutrients* (*n* = 1,038, IF = 4.8), *Plos On*e (*n* = 717, IF = 2.9), *Trends in Endocrinology and Metabolism* (*n* = 635, IF = 11.4) and *Journal of Clinical Investigation* (*n* = 632, IF = 13.3). Table 4 and Figure 3B shows that the most published papers are *Nutrients* (*n* = 47, IF = 4.8), followed by *Plos One* (*n* = 15, IF = 2.9), *Scientific Reports* (*n* = 14, I = 3.8), *Frontiers In Nutrition* (*n* = 11, IF = 4) and *Frontiers In Physiology* (*n* = 10, IF = 3.2). The co-citation journal map shows that *Nutrients*, *Plos One*, *Scientific Reports*, *Frontiers in Physiology* and *Frontiers In Nutrition* are representative centers of collaboration (Figure 4). These findings indicate that the 10 journals listed above have high influence in the field of liver health in KD. Additionally, the data reveal that the number of publications in highly cited journals remains relatively small, and citation intensity in journals with a larger volume of publications is limited. This suggests that research in this field needs to enhance both its breadth and depth.

**Table 3:** Top 10 journals with the most cited journals.

Rank	Journal	ISSN	Cites	Articles	IF (2023)
1	Cell Metabolism	1,550–4,131	1,262	6	27.7
2	Nutrients	2072–6,643	1,038	47	4.8
3	Plos One	1932–6,203	717	15	2.9
4	Trends in Endocrinology and Metabolism	1,043–2,760	635	2	11.4
5	Journal of Clinical Investigation	0021–9,738	632	3	13.3
6	Annual Review of Physiology	0066–4,278	572	1	15.7
7	Nature Reviews Drug Discovery	1,474–1776	437	1	122.7
8	Nature	0028–0836	424	1	50.5
9	Annual Review of Nutrition	0199–9,885	415	1	12.6
10	Scientific Reports	2045–2,322	395	14	3.8

**Table 4:** Top 10 journals with the most published articles.

Rank	Journal	ISSN	Articles	Cites	IF (2023)
1	Nutrients	2072–6,643	47	617	4.8
2	Plos One	1932–6,203	15	527	2.9
3	Scientific Reports	2045–2,322	14	395	3.8
4	Frontiers in Nutrition	2,296-861X	11	82	4
5	Frontiers in Physiology	1,664-042X	10	87	3.2
6	Metabolites	2,218–1989	10	28	3.4
7	Molecular Metabolism	2,212–8,778	10	195	7
8	International Journal of Molecular Sciences	1,422–0067	9	140	4.9
9	Journal of Biological Chemistry	0021–9,258	8	896	4
10	Journal of Inherited Metabolic Disease	0141–8,955	7	150	4.2

### 3.3 Author and co-author

**Figure 5:**
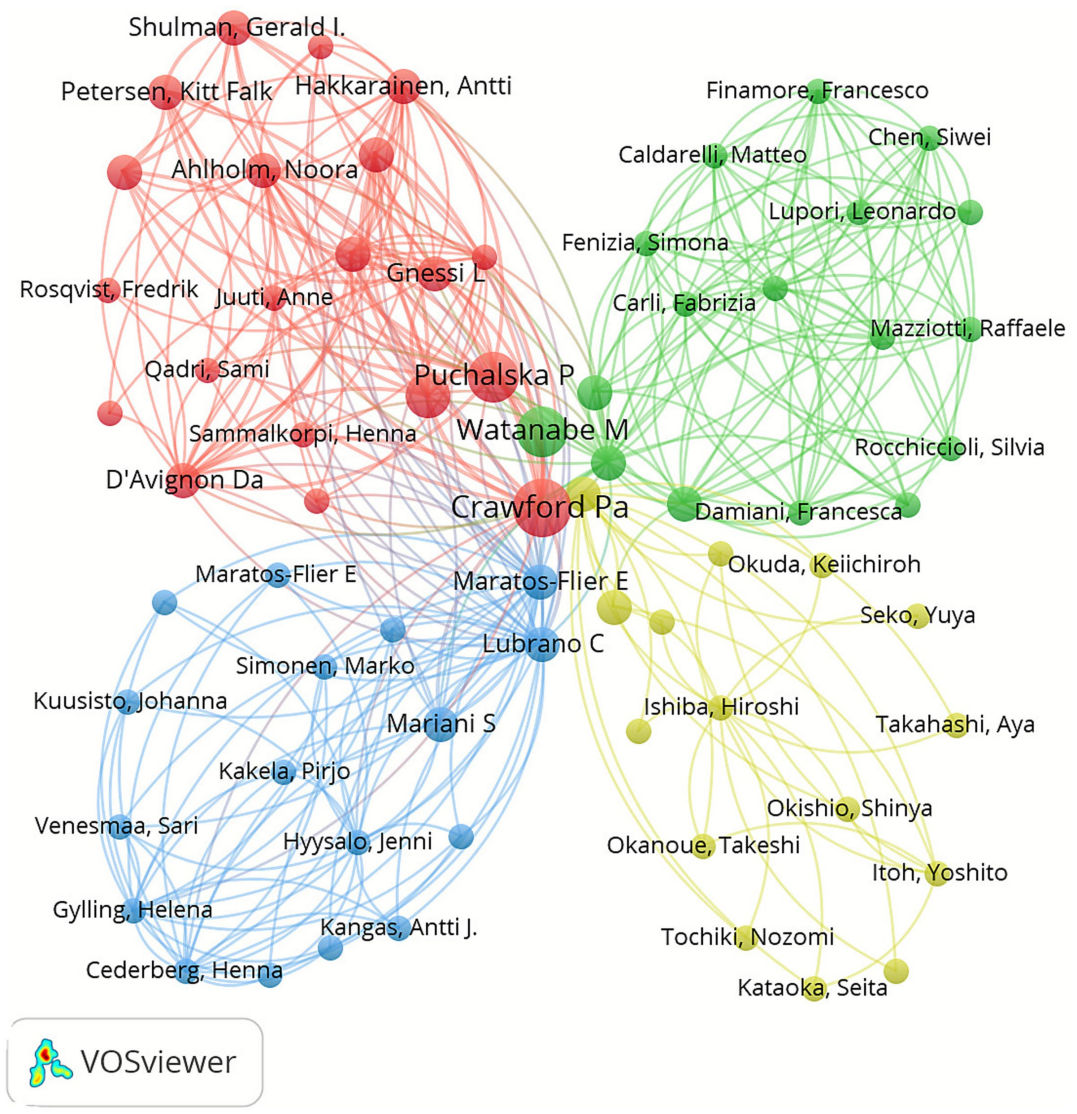
The map of co-authorship in the field of the field of KD in liver health from 2013 to 2024.

Table 5 lists the top 10 authors in terms of the number of published papers. Among them, the top five are Crawford Pa (*n* = 12), Maratos-Flier E (*n* = 9), Watanabe M (*n* = 8), Gnessi L (*n* = 7) and Li J (*n* = 7). In Table 6, we list the top 10 authors who have been cited the most in the literature. The top five authors with the most citations are Crawford Pa (*n* = 174), Puchalska P (*n* = 113), Watanabe M (*n* = 78), Newman Jc (*n* = 74), and Verdin E (*n* = 74). The number of papers published reflects the research productivity of the author to a certain extent, while the number of citations reflects the influence of the author. It is noteworthy that seven researchers, namely Crawford Pa, Watanabe M, Gnessi L, D’Avignon Da, Lubrano C, Mariani S, and Puchalska P, have all appeared in the top 10 in terms of both the number of published papers and the number of citations. This underlines their significant contribution to the field and their status as the most influential authors in the field. It is noteworthy that most of these authors come from the United States and Italy, which highlights the importance and influence of researchers from these two countries in this field. Furthermore, our network map of co-authors (Figure 5) shows a wide and close collaboration among authors, with Crawford Pa, Watanabe M, Gnessi L, D’Avignon Da, Lubrano C, Mariani S, and Puchalska P being the representative centers of collaboration, a finding that highlights their profound influence on the field.

**Table 5:** Top 10 documents authors related to KD in liver health.

Rank	Author	Documents	Citations
1	Crawford Pa	12	174
2	Maratos-Flier E	9	59
3	Watanabe M	8	78
4	Gnessi L	7	69
5	Li J	7	12
6	D’Avignon Da	6	72
7	Liu Y	6	19
8	Lubrano C	6	69
9	Mariani S	6	69
10	Puchalska P	6	113

**Table 6:** Top 10 citations authors related to KD in liver health.

Rank	Author	Citations	Documents
1	Crawford Pa	174	12
2	Puchalska P	113	6
3	Watanabe M	78	8
4	Newman Jc	74	3
5	Verdin E	74	4
6	D’Avignon Da	72	6
7	Basciani S	69	5
8	Gnessi L	69	7
9	Lubrano C	69	6
10	Mariani S	69	6

### 3.4 Most cited references and reference burst

**Figure 6:**
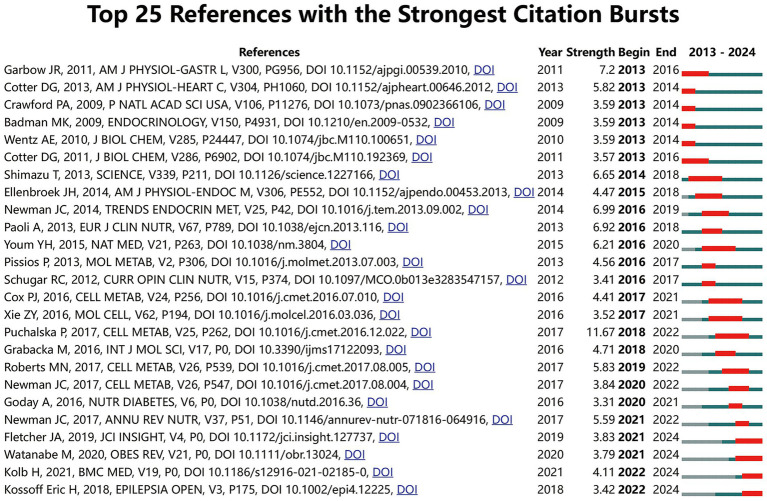
Top 25 references with the strongest citation bursts on the field of KD in liver health.

We identified the top 25 most cited literature in the KD in Liver Health field using the bibliometrix package of R software (Table 7). We found that these literatures have been cited over 120 times, each from 21 different journals, indicating that further research is needed in this field in the future. It is worth noting that among the top 25 references, the journal *Cell Metabolism* has the most but only three articles, which to some extent indicates that the journal *Cell Metabolism* also has a certain influence in this field. However, unfortunately, there is still no dominant journal overall. The top three cited references are “*Multi-dimensional Roles of Ketone Bodies in Fuel Metabolism, Signaling, and Therapeutics*”; “*Ketone bodies as signaling metabolites* “and” *Understanding the Physiology of FGF21* “. However, upon careful examination, we found that these articles only provided a general overview of KD in Liver Health. To determine the most important citation bursts in the KD in Liver Health field, we used CiteSpace (selection criteria: top 25; status number: 2; minimum duration: 2) to obtain a total of 36 articles with the strongest citation highlights, of which 25 are shown in Figure 6. Among them, “*Multidimensional Roles of Ketone Bodies in Fuel Metabolism, Signaling, and Therapeutics*” (Strength: 11.67), “*Hepatic Steam, Inflammation, and ER stress in small maintained long term on a very low carbohydrate ketogenic diet*” (Strength: 7.2), and “*Ketone Bodies as Signaling Metabolism*” (Strength: 6.99) are the top three most cited articles, and interestingly, the most cutting-edge citation explosion are also these three articles. To further understand the research frontiers and hotspots in KD for liver health, we matched the DOIs of the 25 citations in Figure 6 with their titles. Our analysis of highly cited literature reveals that KD has garnered significant attention as a novel dietary therapy due to increasing focus on liver health. However, despite its short-term benefits for certain liver conditions, there is a lack of long-term experimental data on its potential safety risks. Hence, more in-depth research is necessary in this area.

**Table 7:** Top 25 cited references related to KD in liver health.

Paper	DOI	Total citations	TC per Year
Puchalska P, 2017, Cell Metab	10.1016/j.cmet.2016.12.022	888	111
Newman Jc, 2014, Trends Endocrin Met	10.1016/j.tem.2013.09.002	634	57.64
Fisher Fm, 2016, Annu Rev. Physiol	10.1146/annurev-physiol-021115-105339	572	63.56
Cunnane Sc, 2020, Nat Rev. Drug Discov	10.1038/s41573-020-0072-x	437	87.4
Hopkins Bd, 2018, Nature	10.1038/s41586-018-0343-4	424	60.57
Laeger T, 2014, J Clin Invest	10.1172/JCI74915	416	37.82
Newman Jc, 2017, Annu Rev. Nutr	10.1146/annurev-nutr-071816-064916	415	51.88
Ballestri S, 2017, Adv Ther	10.1007/s12325-017-0556-1	349	43.63
Stefan N, 2021, Nat Rev. Endocrinol	10.1038/s41574-020-00462-1	289	72.25
Kim Kh, 2013, Plos One	10.1371/journal.pone.0063517	224	18.67
Hallberg Sj, 2018, Diabetes Ther	10.1007/s13300-018-0373-9	216	30.86
De Lorenzo A, 2019, J Transl Med	10.1186/s12967-019-1919-y	182	30.33
Dushay Jr., 2015, Mol Metab	10.1016/j.molmet.2014.09.008	172	17.2
Athinarayanan Sj, 2019, Front Endocrinol	10.3389/fendo.2019.00348	172	28.67
Cotter Dg, 2014, J Clin Invest	10.1172/JCI76388	147	13.36
Fukao T, 2014, J Inherit Metab Dis	10.1007/s10545-014-9704-9	144	13.09
Dittenhafer-Reed Ke, 2015, Cell Metab	10.1016/j.cmet.2015.03.007	144	14.4
Chriett S, 2019, Sci Rep-Uk	10.1038/s41598-018-36941-9	138	23
Castellana M, 2020, Rev. Endocr Metab Dis	10.1007/s11154-019-09514-y	136	27.2
Shao Ml, 2014, Nat Commun	10.1038/ncomms4528	136	12.36
Luukkonen Pk, 2020, P Natl Acad Sci USA	10.1073/pnas.1922344117	134	26.8
Tognini P, 2017, Cell Metab	10.1016/j.cmet.2017.08.015	128	16
Kosinski C, 2017, Nutrients	10.3390/nu9050517	124	15.5
Ruskin Dn, 2013, Plos One	10.1371/journal.pone.0065021	121	10.08
Bae Hr, 2016, Oncotarget	10.18632/oncotarget.12119	121	13.44

### 3.5 Keyword clusters and evolution

**Figure 7:**
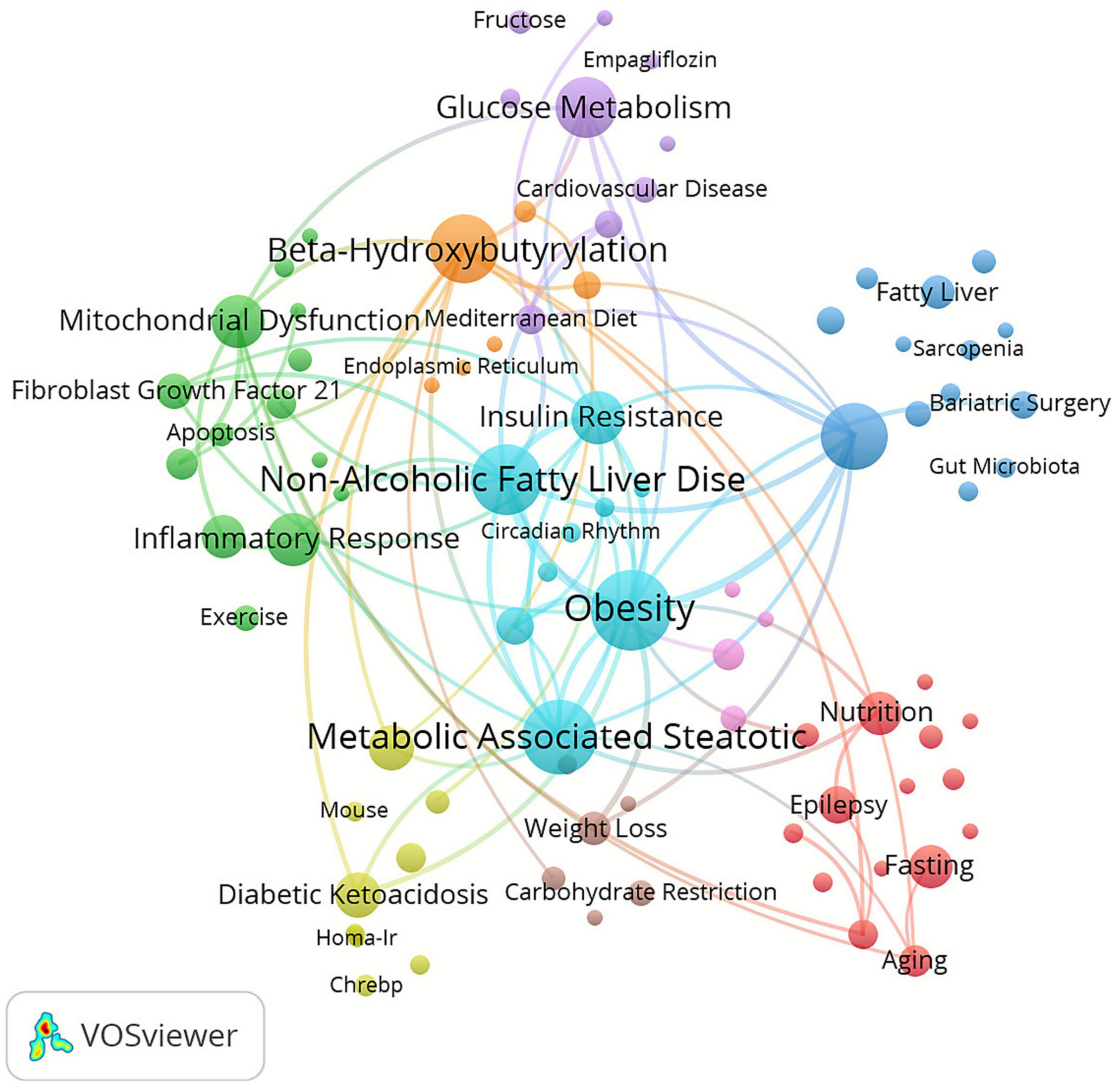
Keywords co-occurrence map of publications on the field of KD in liver health.

Keyword clustering is an effective method for identifying research hotspots and trends in a field. In this study, 88 keywords were extracted using VOSviewer. In this study, a total of 88 keywords were extracted using VOSviewer. Table 8 shows that the top 10 keywords have appeared more than or equal to 19 times. The most frequently occurring keywords are Obesity (*n* = 55), followed by Metabolic Associated Steatotic Liver Disease (*n* = 47), Non-Alcoholic Fatty Liver Disease (*n* = 44), Beta-hydroxybutyrate (*n* = 41), and Low carbohydrate diet (*n* = 38). Then, based on the minimum value of the occurrence of a certain keyword≥3, 88 keywords were selected to draw a keyword clustering graph (Figure 7), in which 9 clusters of different colors were observed. The first cluster (red dots) focuses on the multifaceted impact of diet on health and disease, with a total of 15 keywords including fast, epidemiology, aging, nutrition, Alzheimer & disease, etc. The second cluster (green dots) has 14 keywords, with the theme of diet patterns and health: prevention and treatment of nutritional and metabolic diseases, including mitochondrial dysfunction, inflammatory response, fibroblast growth factor 21, skeletal muscle, exercise, etc. There are 13 keywords in the third cluster (deep blue dots), focusing on the effects of low carbohydrate diet on fatty liver and liver metabolism, including low carbohydrate diet, fatty liver, sarcopenia, bariatric surgery, cirrhosis, etc. The fourth cluster (yellow dot) focuses on the research related to the impact of fatty acid oxidation on metabolic diseases such as diabetes and abnormal lipid metabolism, and contains 12 keywords, including fat acid oxidation, diabetic ketoacidosis, diabetes, dyslipidemia, and hypertensive steatosis. The fifth cluster (purple dots) focuses on nutrition patterns and cardiovascular health, with a total of 9 keywords including glucose metabolism, mediterranean diet, intermittent fasting, cardiovascular disease, fructose, etc. The sixth cluster (light blue dots) focuses on NAFLD, insulin resistance, and novel dietary strategies, with a total of 9 keywords including: obesity, non-alcoholic fatty liver disease, metabolic associated steatotic liver disease, insulin resistance, very low-calorie ketogenic diet, etc. The seventh cluster (orange dots) focuses on the role of beta-hydroxybutyrylation in metabolic regulation, with a total of six keywords including beta-hydroxybutyrylation, adenosine 5′-monophosphate-activated protein kinase, autophagy, endothelial reticulum, pancreatic cancer, etc. The eighth cluster (brown dots) focuses on the effects and responses of carbohydrate restriction on hepatic stellate cells and hepatocytes, with a total of six keywords including: weight less, carbohydrate restriction, acetate, endurance, hepatic stellate cells, etc. The ninth cluster (pink dot) focuses on the effect of controlling keto nutritional diet on type 2 diabetes in animal experiments, and contains four keywords, including: type 2 diabetes mellitus body composition, animal models, nutritional ketosis. In addition, VOSviewer was used to identify 3,207 keywords from Scopus (Supplementary Figure S2). Current research on the KD and liver health converges on several key hotspots: (1) Its clinical application in the management of metabolic and neurological disorders, such as type 2 diabetes and epilepsy. (2) Its regulatory effects on hepatic mitochondrial function, lipid metabolism, and inflammation. (3) Its role in nutritional interventions for obesity and NAFLD, particularly via modulation of the gut–liver axis. (4) Its influence on insulin resistance, hepatic steatosis, fibrosis, and hepatocarcinogenesis through ketone body metabolism and carbohydrate restriction. (5) The molecular adaptations induced by KD, including autophagy, circadian rhythm regulation, and lipid homeostasis.

**Table 8:** Top 10 keywords related to KD in liver health.

Rank	Keyword	Count
1	Obesity	55
2	Metabolic Associated Steatotic Liver Disease	47
3	Non-Alcoholic Fatty Liver Disease	44
4	Beta-hydroxybutyrate	41
5	Low Carbohydrate diet	38
6	Glucose Metabolism	33
7	Mitochondrial Dysfunction	26
8	Inflammatory Response	25
8	Insulin Resistance	25
10	Diabetic Ketoacidation	19
10	Fatty Acid Oxidation	19

**Figure 8:**
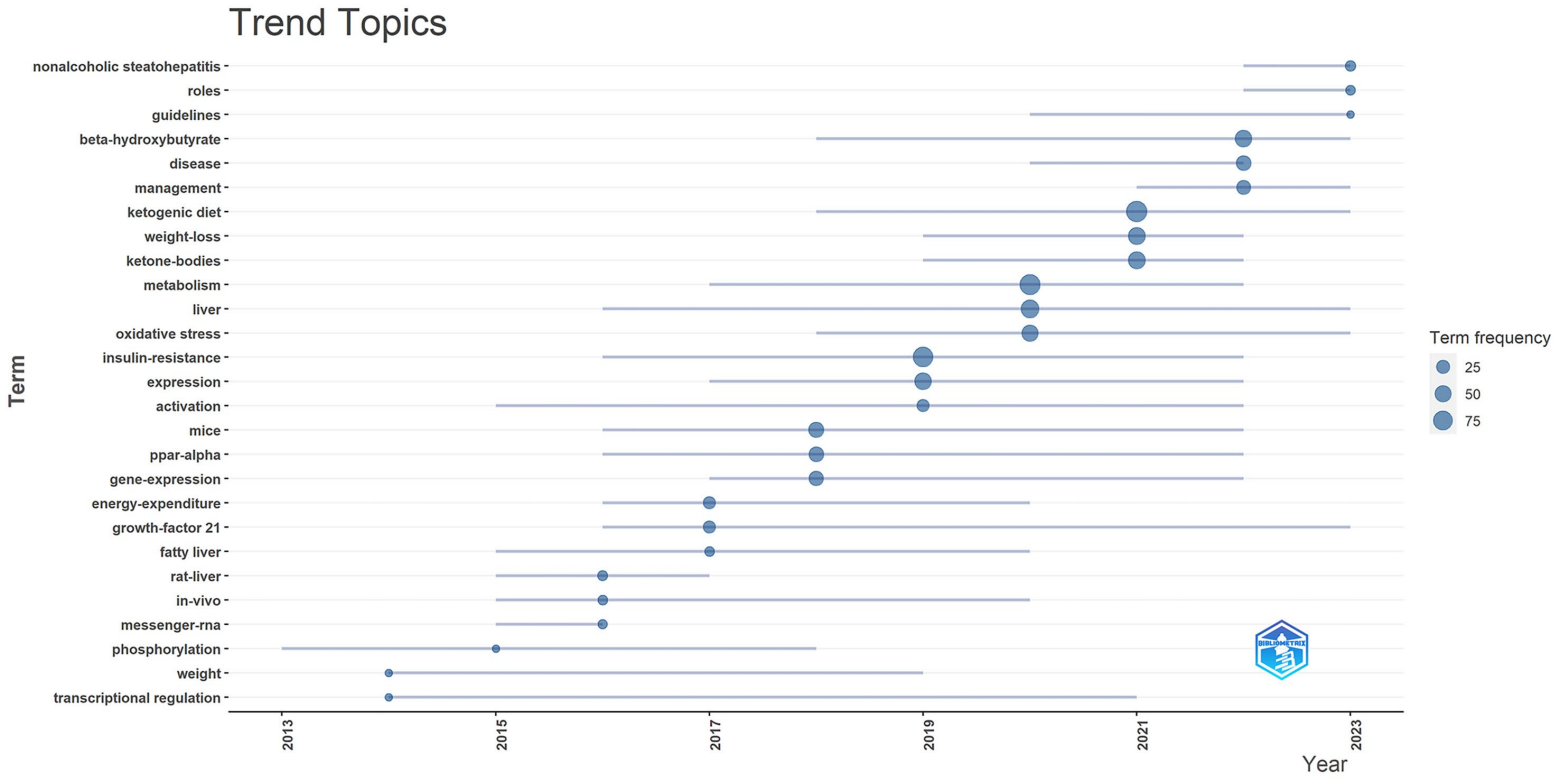
Trend topics on the field of KD in liver health research.

In addition, we generated a trend theme map using the bibliometrix package in R software (Figure 8). Trend theme maps are valuable for tracking the chronological development of specific research topics within a field. They allow us to monitor the evolution of research hotspots over time and gain deeper insights into their progression. By examining the trend map, we analyzed the evolution of research focus and the development trajectory of KD in liver health. Our findings indicate that between 2014 and 2018, the focus was on regulating the expression of fibroblast growth factor 21 (FGF21) through experimental gene editing in mice for the treatment of fatty liver. From 2019 to 2023, the focus shifted to studying weight management guidelines for NAFLD patients, specifically using *β*-hydroxybutyrate (BHB), a ketone body produced through the KD’s metabolic pathway.

Through keyword clustering and evolution analysis, we found that the primary research hotspots in the field of KD and liver health are focused on (1) The impact of KD on the treatment of NAFLD. (2) The impact of KD on the treatment of ALD and cirrhosis. (3) The impact of KD on the treatment of HCC.

## 4 Discussion

### 4.1 General information

To better understand the research focus and trends in KD for liver health, we conducted a bibliometric analysis and data visualization, examining 561 papers published between 2013 and 2024. Our findings reveal a growing trend in publications, particularly between 2019 and 2022, indicating increasing interest in this area. This surge highlights the expanding attention KD has garnered in the study of liver health. Among the countries contributing to this field, the United States published the most papers (*n* = 145), followed by China (*n* = 84) and Italy (*n* = 47). Of the top 10 publishing institutions, five are from the US, two from France, and one each from China and Italy, with Sapienza University of Rome in Italy leading with 11 publications. Notably, seven of the top 10 countries are from Europe and the Americas, while three are from Asia. This suggests that KD-related liver health research is more prominent in Europe and the United States compared to other regions. Interestingly, although the United States leads in the number of publications, there is no single dominant institution in this field. Instead, research is distributed across numerous institutions, suggesting a competitive landscape where many schools are actively engaged. This distribution indicates that research on KD in liver health is still in its early stages, with opportunities to expand the scope and deepen the exploration of this area in future studies. In a study of published journals, we found that 561 research papers were published in 292 journals, important journals include *Nutrients, Plos One, Scientific Reports, Frontiers in Nutrition, Frontiers in Physiology*, and others. Interestingly, *Cell Metabolism* and *Nutrients* are the most cited journals, and *Nutrients* and *Plos One* are the most published journals. The results show that these journals are key publications in the field of KD in Liver Health. It’s worth noting that high-impact journals, such as *Nature Reviews Drug Discovery*, publish fewer articles, yet these articles tend to have a higher citation count. This can be attributed to their rigorous publication standards and significant academic influence. The articles published in these journals often address cutting-edge, interdisciplinary research topics, offering high levels of innovation and theoretical depth. As a result, despite the limited number of publications, each article holds substantial academic value, generating widespread attention and frequent citations in subsequent research. The high citation frequency reflects not only the broad acceptance of the research but also its pivotal role in advancing the field. In our study, we observed that the majority of the top 10 most published and cited authors in the field are from the United States and Italy. Notable figures in this field include Crawford Pa, Puchalska P, Watanabe M, and D’Avignon Da, who have made significant contributions to advancing research on KD and liver health. These researchers play a pivotal role in the development of the field, highlighting the prominent contributions of American and Italian scholars. Current research suggests considerable potential for further growth, making continued and more in-depth investigation crucial for advancing the field, deepening our understanding, and facilitating the translation of findings into clinical practice—ultimately benefiting a broader patient population.

### 4.2 Hotspots and development trends

By analyzing citation frequency, citation explosion, keyword clustering, and keyword trend themes, we have summarized the research hotspots and frontiers in KD in Liver Health as three noteworthy aspects. First, Effects of the KD on Liver Physiology and Pathophysiology. Second, Mechanisms of the KD in Treating Diverse Liver Diseases. Third, Potential Side Effects of the KD on Liver Health.

#### 4.2.1 Effects of the KD on liver physiology and pathophysiology

The KD is a low-carbohydrate, high-fat dietary pattern that forces the body into a state of ketosis, where fat, rather than glucose, is primarily used for energy. This dietary pattern has profound effects on liver physiology and pathophysiology, particularly on lipid metabolism, insulin sensitivity, inflammatory responses, and oxidative stress regulation. The KD not only helps improve liver metabolic function but may also, in certain cases, slow the progression of liver diseases. The following details the effects of the KD on liver physiology and pathophysiology.

##### 4.2.1.1 Promotion of fatty acid oxidation and ketone body production

The core mechanism of the KD is to reduce carbohydrate intake, thereby lowering blood glucose levels and forcing the liver to switch from glucose metabolism to fatty acid oxidation (34). When carbohydrate intake is reduced, the liver breaks down fatty acids through *β*-oxidation to produce ketone bodies, such as β-hydroxybutyrate (BHB) and acetoacetate. These ketones not only provide energy for the liver but also serve as energy sources for other tissues, especially the brain (24).

Under normal conditions, the liver primarily generates glucose through glycogenolysis and gluconeogenesis (35). However, during the KD, the reduced glucose availability shifts the liver’s metabolism toward fatty acid oxidation, generating ketones to meet energy demands. This metabolic switch helps reduce the liver’s dependence on glucose, improves glucose metabolism, and promotes fat burning, thus alleviating hepatic fat accumulation—especially in conditions like NAFLD. This dietary shift may offer therapeutic benefits for these pathological states (36).

##### 4.2.1.2 Improvement of insulin sensitivity and reduction in hepatic fat accumulation

The KD can significantly improve insulin sensitivity, which is particularly relevant in conditions like insulin resistance, NAFLD, and type 2 diabetes. By substantially reducing carbohydrate intake, the diet stabilizes blood glucose levels and decreases insulin secretion, improving insulin sensitivity (37). As insulin levels decrease, the liver’s expression of key enzymes involved in fat synthesis, such as fatty acid synthase (FAS) and acetyl-CoA carboxylase (ACC), is suppressed, reducing fat generation (38).

##### 4.2.1.3 Reduction of liver inflammation and oxidative stress

The KD also plays a crucial role in modulating liver inflammation. Studies have shown that the KD can reduce pro-inflammatory factors in the liver, such as tumor necrosis factor *α* (TNF-α), interleukin-6 (IL-6), and suppress the activation of pro-inflammatory pathways, like nuclear factor kappa B (NF-κB) (39). By inhibiting inflammation, the KD helps slow down liver damage, particularly in conditions related to fatty liver, such as NAFLD (40).

Furthermore, the KD has beneficial effects on oxidative stress regulation. Ketones, especially *β*-hydroxybutyrate, not only serve as an energy source but also exhibit antioxidant properties. Ketones activate the nuclear factor erythroid 2-related factor 2 (NRF2) pathway, which enhances the activity of antioxidant enzymes like superoxide dismutase (SOD) and glutathione peroxidase (GPx), thus helping to neutralize free radicals and mitigate oxidative stress (41).

In liver diseases such as NAFLD and alcoholic fatty liver, oxidative stress and inflammation are common pathological features (42). The KD, by reducing oxidative stress and suppressing inflammation, has the potential to alleviate these pathological features and slow disease progression.

##### 4.2.1.4 Improvement of lipid and bile acid metabolism

The KD alters lipid metabolism, which has significant effects on lipid synthesis and breakdown in the liver (43). During ketone body production, fatty acids are broken down via β-oxidation, generating ketones for energy. This metabolic change helps not only reduce fat accumulation in the liver but also promotes the metabolism of lipids and cholesterol.

Studies have shown that the KD may also regulate the synthesis and metabolism of bile acids, further promoting lipid metabolism (44). The liver is the primary site for bile acid synthesis, and the KD can stimulate bile acid production by modulating the fatty acid levels in the liver, helping to reduce fat deposits in the liver.

##### 4.2.1.5 Potential risks of the KD on liver pathophysiology

Although the KD has positive effects on liver health, long-term adherence to the diet may also pose certain risks to the liver. The high-fat nature of the KD may lead to excessive accumulation of cholesterol and fat-soluble vitamins, potentially increasing the liver’s workload, especially in individuals with pre-existing liver conditions (45).

Additionally, the KD may exert pressure on the gallbladder, particularly in individuals with a history of gallstones or compromised gallbladder function. Excessive fat intake may lead to increased gallbladder activity, raising the risk of gallstone formation (46).

Moreover, prolonged ketone production may impose additional stress on the liver, particularly in individuals with impaired liver metabolism. This can lead to deficiencies in fat-soluble vitamins (such as A, D, E, and K), affecting liver function. Therefore, close monitoring of liver function is necessary when following the KD, especially for extended periods, to ensure safety and long-term effectiveness (47).

The KD exerts multiple beneficial effects on liver physiology by modulating lipid metabolism, improving insulin sensitivity, reducing inflammation, and regulating oxidative stress. It shows potential therapeutic benefits for liver diseases, especially in conditions like fatty liver disease, cirrhosis, and certain metabolic disorders. However, the implementation of the KD should be personalized based on the individual’s condition, and long-term use requires close monitoring to minimize potential risks. By designing appropriate KD plans and working with healthcare providers and nutritionists, individuals can maximize the benefits for liver health while minimizing the potential risks associated with its prolonged use.

#### 4.2.2 Mechanisms of the KD in treating diverse liver diseases

The KD, characterized by a very low carbohydrate intake, moderate protein, and high fats, leads to a metabolic shift in which the body transitions from using glucose to ketone bodies (e.g., acetoacetate, *β*-hydroxybutyrate (BHB), and acetone) as the primary energy source. This alteration in energy metabolism underpins several mechanisms that may prove beneficial in managing a variety of liver disorders, such as NAFLD, alcoholic liver disease (ALD) with early cirrhosis, and HCC.

##### 4.2.2.1 The effect of KD on the treatment of NAFLD

NAFLD primarily results from the accumulation of excessive lipids in the liver, often linked to insulin resistance and metabolic dysfunction (48). Insulin resistance plays a central role in the pathogenesis of NAFLD by promoting lipolysis and leading to the increased influx of free fatty acids into the liver, where they accumulate and cause hepatic steatosis (49). Additionally, insulin resistance exacerbates itself through a feedback loop, which worsens the metabolic disturbances associated with NAFLD (50).

The KD can positively impact insulin resistance by reducing carbohydrate intake, which decreases glucose availability and stabilizes blood sugar levels (51). This reduction in glucose metabolism lowers insulin demand, helping to maintain lower insulin levels, thereby reducing the incidence of insulin-related metabolic disorders in the liver. Furthermore, during ketosis, the liver converts fatty acids into ketone bodies, which provide an alternative energy source to glucose. This shift significantly reduces the liver’s reliance on glucose metabolism, allowing it to oxidize fatty acids more effectively (28).

Insulin is a key regulator of hepatic lipogenesis, promoting fatty acid synthesis through transcription factors such as sterol regulatory element-binding protein 1c (SREBP-1c) (52). Lower insulin levels, facilitated by the KD, decrease the activity of these transcription factors, which in turn reduces the synthesis of triglycerides from fatty acids and acetyl-CoA, thus mitigating excessive lipid accumulation in the liver (53). Additionally, the KD promotes the breakdown of fatty acids into free fatty acids, further contributing to the reduction of liver fat (37).

BHB, one of the primary ketone bodies, has been shown to activate transcription factors like peroxisome proliferator-activated receptor gamma coactivator 1-alpha (PGC-1α), which regulates genes involved in fat metabolism and enhances mitochondrial oxidative capacity (54). By promoting mitochondrial fatty acid oxidation and gene expression related to lipid metabolism, BHB further supports the therapeutic goal of reducing hepatic lipid accumulation (55). However, some studies suggest that the KD may not universally benefit NAFLD, and several factors, including the type and quality of lipids in the diet, the body’s ketogenic reserves, and individual metabolic status, can influence treatment outcomes (56). Research is ongoing to elucidate the full range of effects the KD has on liver lipid metabolism and its potential for managing NAFLD.

It should be noted that in recent years, the term “NAFLD” has gradually been replaced by “metabolic-associated fatty liver disease” (MASLD) (57). The introduction of the term MASLD is a significant advancement in the understanding of this condition, reflecting a more precise classification based on its metabolic associations. While “Non-Alcoholic Fatty Liver Disease” (NAFLD) historically emphasized the exclusion of alcohol consumption, MASLD highlights the disease’s close links with metabolic disorders such as obesity and diabetes. This shift in terminology is necessary as it aligns with the evolving understanding of the disease’s pathophysiology and its connection to metabolic syndrome, offering a more accurate representation of its etiological factors. Furthermore, adopting MASLD in epidemiological research enhances the precision of patient categorization, contributing to better disease stratification and more tailored clinical management. Although the transition from NAFLD to MASLD may pose challenges, particularly regarding historical data, it is a crucial step toward advancing both research and clinical practice.

##### 4.2.2.2 The effect of KD on the treatment of ALD and early liver cirrhosis

Chronic alcohol consumption leads to persistent inflammation, hepatocyte damage, and fibrosis, often progressing to alcoholic fatty liver disease (ALD) and eventually cirrhosis (58). While complete alcohol abstinence remains the primary treatment for ALD and cirrhosis, addressing the metabolic dysfunction in the liver is also crucial. The goal is to reduce hepatic fat accumulation, control inflammation, and mitigate oxidative stress, all of which contribute to liver injury (14).

The KD, with its low carbohydrate content, reduces external glucose availability, encouraging the liver to shift its primary energy source from glucose to fatty acids. This metabolic switch boosts fat oxidation and promotes the breakdown of lipids (59). During ketosis, the liver upregulates metabolic regulators like fibroblast growth factor 21 (FGF21), which facilitates ketone production, particularly BHB (60). BHB plays a significant role in managing alcoholic fatty liver and early cirrhosis by improving insulin sensitivity, reducing hepatic fat accumulation, and providing antioxidant protection against oxidative stress (61). BHB has several mechanisms through which it exerts its beneficial effects. It interacts with G-protein-coupled receptor 109A (GPR109A), triggering anti-inflammatory signaling pathways and suppressing pro-inflammatory pathways mediated by NF-κB (62). This results in reduced cytokine production, such as TNF-*α*, IL-1β, and IL-6, which are key mediators of inflammation. Additionally, BHB enhances the production of anti-inflammatory cytokines like IL-10 and supports the shift of macrophage polarization toward the M2 phenotype, contributing to reduced inflammation and enhanced tissue repair (63). Oxidative stress, a common feature in ALD, is exacerbated by alcohol metabolism, which generates excessive reactive oxygen species (ROS) (64). BHB stabilizes cell membranes and scavenges free radicals, reducing oxidative damage. Moreover, it stimulates the NRF2 pathway, which upregulates antioxidant enzymes like superoxide dismutase (SOD) and glutathione peroxidase (GPx) (65). These enzymes neutralize ROS, reducing further cellular damage. The antioxidant-rich foods typically included in a KD, such as nuts, seeds, and leafy greens, further support the body’s antioxidant defenses, enhancing the diet’s overall effectiveness in mitigating liver damage (66).

In summary, the KD addresses the metabolic disturbances associated with ALD and early cirrhosis by shifting liver metabolism toward enhanced fatty acid oxidation and ketone production. Through the actions of BHB, the diet improves insulin sensitivity, reduces hepatic fat deposition, and exerts anti-inflammatory and antioxidant effects, making it a promising adjunct therapy for managing alcoholic fatty liver disease and early cirrhosis.

##### 4.2.2.3 Reprogramming tumor metabolism in HCC

HCC is a primary form of liver cancer that often develops in the context of chronic liver diseases, including cirrhosis and hepatitis (67). The KD has shown promise in influencing the progression of HCC by altering tumor cell metabolism. Many cancer cells, including those in HCC, rely heavily on glycolysis for energy, even in the presence of oxygen (the Warburg effect). The KD reduces glucose availability, potentially limiting tumor cell growth by depriving them of their preferred energy source (68).

In contrast to normal cells, which can effectively utilize ketone bodies as an alternative energy source, HCC cells struggle to adapt to ketosis due to mitochondrial dysfunction and abnormal ketone metabolism (69). Key enzymes involved in ketone metabolism, such as Alpha-ketoglutarate dehydrogenase and succinyl-CoA transferase, are often less active in tumor cells, preventing efficient utilization of ketones. This metabolic disruption inhibits tumor growth and proliferation, offering a potential therapeutic benefit (70).

Additionally, the increased production of ketone bodies like *β*-hydroxybutyrate under a KD has been shown to enhance oxidative stress in tumor cells (71). While ketones reduce ROS production in normal cells, tumor cells, with their impaired mitochondria, experience heightened oxidative stress under a KD (72). This stress may promote apoptosis and autophagy in tumor cells, contributing to tumor reduction. The KD can also inhibit angiogenesis, the process by which tumors develop blood vessels to supply nutrients, further limiting tumor growth (73).

The KD’s ability to alter the metabolic environment of HCC cells, enhance oxidative stress, and potentially sensitize tumors to chemotherapy or radiotherapy makes it a promising adjunct treatment (69). Studies have shown that combining a KD with other therapeutic strategies, such as small molecule inhibitors or statins, may synergistically enhance treatment outcomes. These treatments work by inhibiting cancer cell growth and metabolism while promoting tumor cell apoptosis (74).

In summary, the KD’s impact on tumor cell metabolism, its ability to increase oxidative stress in cancer cells, and its potential to improve the effectiveness of conventional therapies make it a promising adjunctive treatment for HCC.

#### 4.2.3 Potential side effects of the KD on liver health

While the KD has shown positive effects in improving liver metabolism, reducing fat accumulation, and combating certain liver diseases such as NAFLD and ALD, its potential side effects on liver health should not be overlooked. Long-term adherence to the KD may trigger negative effects, particularly in individuals with pre-existing liver dysfunction or underlying liver diseases. The following section explores the potential adverse effects of the KD on the liver.

##### 4.2.3.1 Increased liver fat accumulation

One of the key therapeutic benefits of the KD is its ability to reduce liver fat accumulation, especially in cases of NAFLD. By limiting carbohydrate intake and shifting the body’s energy reliance from glucose to fats, the KD encourages fat oxidation and ketone production. However, improper fat selection and excessive fat intake can exacerbate fat accumulation in the liver, especially if the liver has not yet fully adapted to the ketogenic state. This can lead to an aggravation of hepatic steatosis (fatty liver), particularly in individuals with pre-existing liver damage (75).

Studies suggest that in some cases, the KD may induce metabolic dysregulation in liver fatty acid metabolism, causing an accumulation of triglycerides and free fatty acids within hepatocytes (76). This issue may be particularly dangerous for individuals who already have compromised liver function, as the excess lipid deposition can further impair liver function and accelerate liver injury. Additionally, the composition of fats in the diet is crucial; if the diet includes high levels of unhealthy fats, it may not only contribute to increased liver fat but could also potentially elevate the risk of liver fibrosis and other complications over time (77).

##### 4.2.3.2 Increased metabolic burden on the liver

The KD imposes a significant metabolic burden on the liver, particularly in individuals whose liver function is already compromised (78). During ketosis, the liver plays a central role in metabolizing fatty acids and converting them into ketone bodies for energy. This metabolic shift requires the liver to accelerate the oxidation of fatty acids, which can increase the liver’s workload.

For individuals with pre-existing liver disease or reduced hepatic function, this increased metabolic stress may lead to further liver damage. Chronic, excessive oxidative stress and the increased demand for fatty acid metabolism can strain hepatocytes, potentially leading to liver dysfunction or even liver failure in extreme cases (79). Moreover, the accumulation of ketone bodies, while beneficial in many instances, could overwhelm liver detoxification mechanisms in individuals with liver disease, complicating the clinical picture and potentially exacerbating hepatic injury (80).

##### 4.2.3.3 Elevated cholesterol levels and increased risk of gallstones

Another potential side effect of the KD is an increase in cholesterol levels, which could contribute to the formation of gallstones (81). The high-fat nature of the diet leads to increased cholesterol synthesis in the liver and increased secretion of cholesterol into the bile. Although bile acids play a crucial role in the digestion and absorption of fats, excessive cholesterol levels can overwhelm the liver’s ability to process and excrete it via bile (82).

For individuals with pre-existing high cholesterol or those who have a history of gallstones, the KD could pose an additional risk by promoting the formation of new gallstones (83). The high intake of dietary fats, particularly saturated fats, may increase the risk of bile becoming supersaturated with cholesterol, which is a known precursor to gallstone formation. Therefore, individuals with a history of gallstones or those at risk should be cautious when considering the KD, as it may exacerbate their condition.

##### 4.2.3.4 Potential nutrient deficiencies

The strict nature of the KD, which severely restricts carbohydrate intake and emphasizes fats and proteins, could lead to certain nutrient deficiencies over time. While the diet can be rich in vitamins A, D, E, and K due to its high-fat content, it may lack essential nutrients found in carbohydrate-rich foods, such as certain B vitamins, fiber, and antioxidants (84). The lack of these nutrients can potentially lead to gastrointestinal issues, muscle cramps, or even bone health problems due to insufficient vitamin D and calcium (85).

Additionally, certain liver diseases, such as cirrhosis, can already compromise the liver’s ability to process and store essential nutrients. In these cases, the KD may exacerbate nutrient imbalances, further impairing liver function and overall health. Therefore, careful monitoring of nutrient levels and supplementation may be required for individuals with liver disease following a KD (86).

##### 4.2.3.5 Increased risk of liver inflammation in certain individuals

While the KD has anti-inflammatory properties for many individuals, it may have the opposite effect in others, especially those with compromised liver function. In individuals with existing liver inflammation, such as those with ALD or autoimmune hepatitis, the KD may initially exacerbate liver inflammation, especially during the early phases of adaptation. This could lead to an increase in liver enzymes, liver swelling, or discomfort (87).

Furthermore, if the KD is not well-balanced in terms of fat types or if the intake of inflammatory fats is high, it could lead to an inflammatory response within the liver, potentially aggravating liver conditions and impairing liver function. Careful attention to the quality of fats in the diet is essential to mitigate this risk, especially in individuals with underlying liver pathology (88).

While the KD offers significant therapeutic potential for liver diseases such as NAFLD and ALD, it is essential to consider the potential side effects on liver health. These include the increased risk of liver fat accumulation, elevated metabolic burden on the liver, higher cholesterol levels, nutrient deficiencies, and exacerbated liver inflammation. As such, the KD should be approached with caution, particularly in individuals with pre-existing liver dysfunction or other metabolic conditions. It is highly recommended that individuals considering or currently following the KD do so under the supervision of a healthcare professional to ensure safe and effective implementation and to monitor for any potential adverse effects.

### 4.3 Limitations

This article is intended to enhance understanding of the development and current research hotspots in the field of KD in liver health, and to explore potential research directions of significant value. However, we acknowledge some limitations in our study. Firstly, our research relies solely on data from the WoSCC database and Scopus database, which may lead to the omission of articles published exclusively in other databases. Nevertheless, the WoSCC database and Scopus database are widely regarded for its high-quality and credible sources, making it a reasonable choice for our bibliometric analysis. Secondly, we only analyzed English publications, which may lead to the neglect of publications in other languages and result in source bias. In addition, in terms of article types, we only included reviews and Articles for analysis, which might also lead to data omissions. Despite these limitations, our research offers a comprehensive overview of the field, highlighting key topics and trends. By considering these factors, researchers can gain insights into the field’s overall development, identify potential research directions, and use this information to guide future exploration.

## 5 Conclusion

Our research clearly highlights the key research hotspots and emerging frontiers in KD related to liver health. Below is a summary of the key knowledge areas and research trends in this field:

- The field of KD has attracted researchers from around the world studying liver health, with the United States, China, and Italy being the most active countries. With the deepening of KD in Liver Health research in the future, cooperation between countries will continue to be close.
- Among cancer-related literature, Nutrients has the most publications and Cell Metabolism has the most citations. Nutrients and Cell Metabolism are representative journals in the field of KD in Liver Health research.
- The co-authors of KD in Liver Health revealed extensive and close collaboration among authors, represented by Crawford Pa, Watanabe M, and Gnessi L.
- The impact of KD on the treatment of NAFLD has become a hot topic and trend in the field of liver health research.
- The impact of KD on the treatment of ALD and cirrhosis has become a hot topic and trend in the field of liver health research.
- The impact of KD on the treatment of HCC has become a hot topic and trend in the field of liver health research.

In summary, our research offers valuable insights into the trends and hotspots of KD in liver health. These findings can help researchers quickly grasp the state of the field and identify new directions for future exploration. By highlighting the limitations and potential focus areas of current research, our study provides guidance for researchers to deepen their investigations and encourages them to explore innovative avenues in their work.

## Data Availability

Publicly available datasets were analyzed in this study. This data can be found at: https://www.webofscience.com.

## References

[1] Devarbhavi H Asrani SK Arab JP Nartey YA Pose E Kamath PS. Global burden of liver disease: 2023 update. J Hepatol. (2023) 79:516–37. doi: 10.1016/j.jhep.2023.03.017, 36990226

[2] Han S Yang Z Zhang T Ma J Chandler K Liangpunsakul S. Epidemiology of alcohol-associated liver disease. Clin Liver Dis. (2021) 25:483–92. doi: 10.1016/j.cld.2021.03.009, 34229835 PMC8996817

[3] Manikat R Ahmed A Kim D. Up-to-date global epidemiology of nonalcoholic fatty liver disease. Hepatobiliary Surg Nutr. (2023) 12:956–9. doi: 10.21037/hbsn-23-548, 38115930 PMC10727827

[4] Singh SP Madke T Chand P. Global epidemiology of hepatocellular carcinoma. J Clin Exp Hepatol. (2025) 15:102446. doi: 10.1016/j.jceh.2024.102446, 39659901 PMC11626783

[5] Storandt MH Tella SH Wieczorek MA Hodge D Elrod JK Rosenberg PS . Projected incidence of hepatobiliary cancers and trends based on age, race, and gender in the United States. Cancer. (2024) 16:684. doi: 10.3390/cancers16040684, 38398075 PMC10886529

[6] Muzurović E Mikhailidis DP Mantzoros C. Non-alcoholic fatty liver disease, insulin resistance, metabolic syndrome and their association with vascular risk. Metabolism. (2021) 119:154770. doi: 10.1016/j.metabol.2021.154770, 33864798

[7] Ginès P Krag A Abraldes JG Solà E Fabrellas N Kamath PS. Liver cirrhosis. Lancet. (2021) 398:1359–76. doi: 10.1016/S0140-6736(21)01374-X, 34543610

[8] Stefano JT Duarte SMB Ribeiro Leite Altikes RG Oliveira CP. Non-pharmacological management options for MAFLD: a practical guide. Ther Adv Endocrinol Metab. (2023) 14:20420188231160394. doi: 10.1177/20420188231160394, 36968655 PMC10031614

[9] Caturano A Galiero R Loffredo G Vetrano E Medicamento G Acierno C . Effects of a combination of empagliflozin plus metformin vs. metformin monotherapy on NAFLD progression in type 2 diabetes: the IMAGIN pilot study. Biomedicine. (2023) 11:322. doi: 10.3390/biomedicines11020322, 36830859 PMC9952909

[10] Wang MY Prabahar K Găman MA Zhang JL. Vitamin E supplementation in the treatment on nonalcoholic fatty liver disease (NAFLD): evidence from an umbrella review of meta-analysis on randomized controlled trials. J Dig Dis. (2023) 24:380–9. doi: 10.1111/1751-2980.13210, 37503812

[11] Patel Chavez C Cusi K Kadiyala S. The emerging role of glucagon-like peptide-1 receptor agonists for the management of NAFLD. J Clin Endocrinol Metabol. (2022) 107:29–38. doi: 10.1210/clinem/dgab578, 34406410 PMC8684453

[12] Mantovani A Byrne CD Targher G. Efficacy of peroxisome proliferator-activated receptor agonists, glucagon-like peptide-1 receptor agonists, or sodium-glucose cotransporter-2 inhibitors for treatment of non-alcoholic fatty liver disease: a systematic review. Lancet Gastroenterol Hepatol. (2022) 7:367–78. doi: 10.1016/S2468-1253(21)00261-2, 35030323

[13] Kim YJ Kim HJ Lee SG Jang SI Go HS Lee WJ . Aerobic exercise for eight weeks provides protective effects towards liver and cardiometabolic health and adipose tissue remodeling under metabolic stress for one week: a study in mice. Metabolism. (2022) 130:155178. doi: 10.1016/j.metabol.2022.155178, 35227728

[14] Singal AK Mathurin P. Diagnosis and treatment of alcohol-associated liver disease: a review. JAMA. (2021) 326:165–76. doi: 10.1001/jama.2021.7683, 34255003

[15] Torres S Hardesty J Barrios M Garcia-Ruiz C Fernandez-Checa JC Singal AK. Mitochondria and alcohol-associated liver disease: pathogenic role and target for therapy. In: Seminars in liver disease. New York, NY, USA: Thieme Medical Publishers, Inc. (2024) 10.1055/a-2421-5658 PMC12981299 39317216

[16] Yoshiji H Nagoshi S Akahane T Asaoka Y Ueno Y Ogawa K . Evidence-based clinical practice guidelines for liver cirrhosis 2020. J Gastroenterol. (2021) 56:593–619. doi: 10.1007/s00535-021-01788-x, 34231046 PMC8280040

[17] Aithal GP Palaniyappan N China L Härmälä S Macken L Ryan JM . Guidelines on the management of ascites in cirrhosis. Gut. (2021) 70:9–29. doi: 10.1136/gutjnl-2020-321790, 33067334 PMC7788190

[18] Jachs M Reiberger T. Prevention of variceal bleeding and rebleeding by nonselective beta-blockers: a tailored approach. Clin Liver Dis. (2021) 25:311–26. doi: 10.1016/j.cld.2021.01.004, 33838852

[19] Chien R-N Liaw Y-F. Current trend in antiviral therapy for chronic hepatitis B. Viruses. (2022) 14:434. doi: 10.3390/v14020434, 35216027 PMC8877417

[20] Wazir H Abid M Essani B Saeed H Khan MA Nasrullah F . Diagnosis and treatment of liver disease: current trends and future directions. Cureus. (2023) 15. doi: 10.7759/cureus.49920, 38174191 PMC10763979

[21] Mak L-Y Liu K Chirapongsathorn S Yew KC Tamaki N Rajaram RB . Liver diseases and hepatocellular carcinoma in the Asia-Pacific region: burden, trends, challenges and future directions. Nat Rev Gastroenterol Hepatol. (2024) 21:834–51. doi: 10.1038/s41575-024-00967-4, 39147893

[22] Vogel A Qin S Kudo M Su Y Hudgens S Yamashita T . Lenvatinib versus sorafenib for first-line treatment of unresectable hepatocellular carcinoma: patient-reported outcomes from a randomised, open-label, non-inferiority, phase 3 trial. Lancet Gastroenterol Hepatol. (2021) 6:649–58. doi: 10.1016/S2468-1253(21)00110-2, 34087115

[23] Bao MH-R Wong CC-L. Hypoxia, metabolic reprogramming, and drug resistance in liver cancer. Cells. (2021) 10. doi: 10.3390/cells10071715, 34359884 PMC8304710

[24] Zhu H Bi D Zhang Y Kong C Du J Wu X . Ketogenic diet for human diseases: the underlying mechanisms and potential for clinical implementations. Signal Transduct Target Ther. (2022) 7:11. doi: 10.1038/s41392-021-00831-w, 35034957 PMC8761750

[25] Battezzati A Foppiani A Leone A De Amicis R Spadafranca A Mari A . Acute insulin secretory effects of a classic ketogenic meal in healthy subjects: a randomized cross-over study. Nutrients. (2023) 15:1119. doi: 10.3390/nu15051119, 36904127 PMC10005334

[26] Zhao X An X Yang C Sun W Ji H Lian F. The crucial role and mechanism of insulin resistance in metabolic disease. Front Endocrinol. (2023) 14:1149239. doi: 10.3389/fendo.2023.1149239, 37056675 PMC10086443

[27] Gao L Chen X Fu Z Yin J Wang Y Sun W . Kinsenoside alleviates alcoholic liver injury by reducing oxidative stress, inhibiting endoplasmic reticulum stress, and regulating AMPK-dependent autophagy. Front Pharmacol. (2022) 12:747325. doi: 10.3389/fphar.2021.747325, 35115920 PMC8804359

[28] Puchalska P Crawford PA. Metabolic and signaling roles of ketone bodies in health and disease. Annu Rev Nutr. (2021) 41:49–77. doi: 10.1146/annurev-nutr-111120-111518, 34633859 PMC8922216

[29] Ferrere G Alou MT Liu P Goubet A-G Fidelle M Kepp O . Ketogenic diet and ketone bodies enhance the anticancer effects of PD-1 blockade. JCI Insight. (2021) 6:e145207. doi: 10.1172/jci.insight.145207, 33320838 PMC7934884

[30] Lan Y Jin C Kumar P Yu X Lenahan C Sheng J. Ketogenic diets and hepatocellular carcinoma. Front Oncol. (2022) 12:879205. doi: 10.3389/fonc.2022.879205, 35600387 PMC9115558

[31] Cione E Abrego Guandique DM Caroleo MC Luciani F Colosimo M Cannataro R. Liver damage and microRNAs: an update. Curr Issues Mol Biol. (2022) 45:78–91. doi: 10.3390/cimb45010006, 36661492 PMC9857663

[32] Cannataro R Perri M Gallelli L Caroleo MC De Sarro G Cione E. Ketogenic diet acts on body remodeling and microRNAs expression profile. MicroRNA. (2019) 8:116–26. doi: 10.2174/2211536608666181126093903, 30474543

[33] Kumar M George RJ Ps A. Bibliometric analysis for medical research. Indian J Psychol Med. (2023) 45:277–82. doi: 10.1177/0253717622110361 37152388 PMC10159556

[34] McGaugh E Barthel B. A review of ketogenic diet and lifestyle. Mo Med. (2022) 119:84. doi: 10.1111/j.1528-1167.2008.01821.x, 36033148 PMC9312449

[35] Nogueira-Ferreira R Oliveira PF Ferreira R. Liver metabolism: the pathways underlying glucose utilization and production. In: Ferreira R Oliveira PF Nogueira-Ferreira R editors. Glycolysis. Amsterdam, Netherlands: Elsevier (2024). 141–56.

[36] Ahmad Y Seo DS Jang Y. Metabolic effects of ketogenic diets: exploring whole-body metabolism in connection with adipose tissue and other metabolic organs. Int J Mol Sci. (2024) 25:7076. doi: 10.3390/ijms25137076, 39000187 PMC11241756

[37] Jani S Da Eira D Stefanovic M Ceddia RB. The ketogenic diet prevents steatosis and insulin resistance by reducing lipogenesis, diacylglycerol accumulation and protein kinase C activity in male rat liver. J Physiol. (2022) 600:4137–51. doi: 10.1113/JP283552, 35974660

[38] Li M Chi X Wang Y Setrerrahmane S Xie W Xu H. Trends in insulin resistance: insights into mechanisms and therapeutic strategy. Signal Transduct Target Ther. (2022) 7:216. doi: 10.1038/s41392-022-01073-0, 35794109 PMC9259665

[39] Srivastava S Pawar VA Tyagi A Sharma KP Kumar V Shukla SK. Immune modulatory effects of ketogenic diet in different disease conditions. Ther Immunol. (2022) 3:1–15. doi: 10.3390/immuno3010001

[40] Long F Bhatti MR Kellenberger A Sun W Modica S Höring M . A low-carbohydrate diet induces hepatic insulin resistance and metabolic associated fatty liver disease in mice. Mol Metab. (2023) 69:101675. doi: 10.1016/j.molmet.2023.101675, 36682412 PMC9900440

[41] Paoli A Cerullo G. Investigating the link between ketogenic diet, NAFLD, mitochondria, and oxidative stress: a narrative review. Antioxidants. (2023) 12:1065. doi: 10.3390/antiox12051065, 37237931 PMC10215390

[42] Delli Bovi AP Marciano F Mandato C Siano MA Savoia M Vajro P. Oxidative stress in non-alcoholic fatty liver disease. An updated mini review. Front Med. (2021) 8:595371. doi: 10.3389/fmed.2021.595371, 33718398 PMC7952971

[43] Yang Z Mi J Wang Y Xue L Liu J Fan M . Effects of low-carbohydrate diet and ketogenic diet on glucose and lipid metabolism in type 2 diabetic mice. Nutrition. (2021) 89:111230. doi: 10.1016/j.nut.2021.111230, 33838492

[44] Patel MJ Jones A Jiang Y Gowda P VanWagner LB Cotter TG . Psychiatric disorders in patients with hepatocellular carcinoma: a large US cohort of commercially insured individuals. Aliment Pharmacol Ther. (2024) 60:469–78. doi: 10.1111/apt.18115, 38863242 PMC12277950

[45] Shalabi H Alotaibi A Alqahtani A Alattas H Alghamdi Z Alattas SHK . Ketogenic diets: side effects, attitude, and quality of life. Cureus. (2021) 13:e20390. doi: 10.7759/cureus.20390 35036220 PMC8752375

[46] Choi J Young TL Chartier LB. Recurrent acute pancreatitis during a ketogenic diet—a case report and literature review. Int J Emerg Med. (2021) 14:1–5. doi: 10.1186/s12245-021-00374-5, 34525949 PMC8444592

[47] Kolb H Kempf K Röhling M Lenzen-Schulte M Schloot NC Martin S. Ketone bodies: from enemy to friend and guardian angel. BMC Med. (2021) 19:1–15. doi: 10.1186/s12916-021-02185-0, 34879839 PMC8656040

[48] Pouwels S Sakran N Graham Y Leal A Pintar T Yang W . Non-alcoholic fatty liver disease (NAFLD): a review of pathophysiology, clinical management and effects of weight loss. BMC Endocr Disord. (2022) 22:63. doi: 10.1186/s12902-022-00980-1, 35287643 PMC8919523

[49] Sakurai Y Kubota N Yamauchi T Kadowaki T. Role of insulin resistance in MAFLD. Int J Mol Sci. (2021) 22:4156. doi: 10.3390/ijms22084156, 33923817 PMC8072900

[50] Szukiewicz D. Molecular mechanisms for the vicious cycle between insulin resistance and the inflammatory response in obesity. Int J Mol Sci. (2023) 24:9818. doi: 10.3390/ijms24129818, 37372966 PMC10298329

[51] Napoleão A Fernandes L Miranda C Marum AP. Effects of calorie restriction on health span and insulin resistance: classic calorie restriction diet vs. ketosis-inducing diet. Nutrients. (2021) 13. doi: 10.3390/nu13041302, 33920973 PMC8071299

[52] Ferré P Phan F Foufelle F. SREBP-1c and lipogenesis in the liver: an update. Biochem J. (2021) 478:3723–39. doi: 10.1042/BCJ20210071, 34673919

[53] Fernandes GW Bocco BM. Hepatic mediators of lipid metabolism and ketogenesis: focus on fatty liver and diabetes. Curr Diabetes Rev. (2021) 17:81–92. doi: 10.2174/1573399816999201103141216 33143628

[54] Wells RG Neilson LE McHill AW Hiller AL. Dietary fasting and time-restricted eating in Huntington’s disease: therapeutic potential and underlying mechanisms. Transl Neurodegener. (2024) 13:17. doi: 10.1186/s40035-024-00406-z, 38561866 PMC10986006

[55] Pan A Sun X-M Huang F-Q Liu J-F Cai Y-Y Wu X . The mitochondrial β-oxidation enzyme HADHA restrains hepatic glucagon response by promoting β-hydroxybutyrate production. Nat Commun. (2022) 13:386. doi: 10.1038/s41467-022-28044-x, 35046401 PMC8770464

[56] Katsiki N Stoian AP Rizzo M. Dietary patterns in non-alcoholic fatty liver disease (NAFLD): stay on the straight and narrow path! Clínica e Investigación en Arteriosclerosis (English Edition). (2022) 34:23–30. doi: 10.1016/j.artere.2022.07.002, 35131122

[57] Rinella ME Sookoian S. From NAFLD to MASLD: updated naming and diagnosis criteria for fatty liver disease. J Lipid Res. (2024) 65:100485. doi: 10.1016/j.jlr.2023.100485, 38103785 PMC10824973

[58] Wu X Fan X Miyata T Kim A Cajigas-Du Ross CK Ray S . Recent advances in understanding of pathogenesis of alcohol-associated liver disease. Annu Rev Pathol Mech Dis. (2023) 18:411–38. doi: 10.1146/annurev-pathmechdis-031521-030435, 36270295 PMC10060166

[59] Ashtary-Larky D Bagheri R Bavi H Baker JS Moro T Mancin L . Ketogenic diets, physical activity and body composition: a review. Br J Nutr. (2022) 127:1898–920. doi: 10.1017/S0007114521002609, 34250885 PMC9244428

[60] Li J He W Wu Q Qin Y Luo C Dai Z . Ketogenic diets and β-hydroxybutyrate in the prevention and treatment of diabetic kidney disease: current progress and future perspectives. BMC Nephrol. (2025) 26:1–14. doi: 10.1186/s12882-025-04019-0, 40055596 PMC11887203

[61] Petagine L Zariwala MG Patel VB. Non-alcoholic fatty liver disease: immunological mechanisms and current treatments. World J Gastroenterol. (2023) 29:4831–50. doi: 10.3748/wjg.v29.i32.4831, 37701135 PMC10494768

[62] Qi J Yang Q Xia Q Huang F Guo H Cui H . Low glucose plus β-Hydroxybutyrate induces an enhanced inflammatory response in yak alveolar macrophages via activating the GPR109A/NF-κB signaling pathway. Int. J. Mol. Sci. (2023) 24:11331. 37511091 10.3390/ijms241411331 PMC10379377

[63] Huang C Wang J Liu H Huang R Yan X Song M . Ketone body β-hydroxybutyrate ameliorates colitis by promoting M2 macrophage polarization through the STAT6-dependent signaling pathway. BMC Med. (2022) 20:148. doi: 10.1186/s12916-022-02352-x, 35422042 PMC9011974

[64] Yang YM Cho YE Hwang S. Crosstalk between oxidative stress and inflammatory liver injury in the pathogenesis of alcoholic liver disease. Int J Mol Sci. (2022) 23:774. doi: 10.3390/ijms23020774, 35054960 PMC8775426

[65] Pei J Pan X Wei G Hua Y. Research progress of glutathione peroxidase family (GPX) in redoxidation. Front Pharmacol. (2023) 14:1147414. doi: 10.3389/fphar.2023.1147414, 36937839 PMC10017475

[66] Dixit V Joseph Kamal SW Bajrang Chole P Dayal D Chaubey KK Pal AK . Functional foods: exploring the health benefits of bioactive compounds from plant and animal sources. J Food Qual. (2023) 2023:1–22. doi: 10.1155/2023/5546753, 41281681

[67] Papatheodoridi A Papatheodoridis G. Hepatocellular carcinoma: the virus or the liver? Liver Int. (2023) 43:22–30. doi: 10.1111/liv.15253, 35319167

[68] Barrea L Caprio M Tuccinardi D Moriconi E Di Renzo L Muscogiuri G . Could ketogenic diet “starve” cancer? Emerging evidence. Crit Rev Food Sci Nutr. (2022) 62:1800–21. doi: 10.1080/10408398.2020.1847030, 33274644

[69] Qian L Li Y Cao Y Meng G Peng J Li H . Pan-cancer analysis of glycolytic and ketone bodies metabolic genes: implications for response to ketogenic dietary therapy. Front Oncol. (2021) 11:689068. doi: 10.3389/fonc.2021.689068, 34692477 PMC8529115

[70] NOTEREF _Ref216089723 \f ^1^Bornstein R Mulholland MT Sedensky M Morgan P Johnson SC. Glutamine metabolism in diseases associated with mitochondrial dysfunction. Mol Cell Neurosci. (2023) 126:103887. doi: 10.1016/j.mcn.2023.103887, 37586651 PMC10773532

[71] Alhamzah SA Gatar OM Alruwaili NW. Effects of ketogenic diet on oxidative stress and cancer: a literature review. Adv Cancer Biol Metastasis. (2023) 7:100093. doi: 10.1016/j.adcanc.2023.100093

[72] Chimienti G Orlando A Lezza AMS D’Attoma B Notarnicola M Gigante I . The ketogenic diet reduces the harmful effects of stress on gut mitochondrial biogenesis in a rat model of irritable bowel syndrome. Int J Mol Sci. (2021) 22:3498. doi: 10.3390/ijms22073498, 33800646 PMC8037144

[73] Talib WH Mahmod AI Kamal A Rashid HM Alashqar AM Khater S . Ketogenic diet in cancer prevention and therapy: molecular targets and therapeutic opportunities. Curr Issues Mol Biol. (2021) 43:558–89. doi: 10.3390/cimb43020042, 34287243 PMC8928964

[74] Yang L TeSlaa T Ng S Nofal M Wang L Lan T . Ketogenic diet and chemotherapy combine to disrupt pancreatic cancer metabolism and growth. Med. (2022) 3:119–136. 35425930 10.1016/j.medj.2021.12.008 PMC9004683

[75] Li Y Yang X Zhang J Jiang T Zhang Z Wang Z . Ketogenic diets induced glucose intolerance and lipid accumulation in mice with alterations in gut microbiota and metabolites. MBio. (2021) 12:03601–20. doi: 10.1128/mBio.03601-20, 33785628 PMC8092315

[76] Schutz Y Montani JP Dulloo AG. Low-carbohydrate ketogenic diets in body weight control: a recurrent plaguing issue of fad diets? Obes Rev. (2021) 22:e13195. doi: 10.1111/obr.13195, 33471427

[77] Wernicke C Pohrt A Pletsch-Borba L Apostolopoulou K Hornemann S Meyer N . Effect of unsaturated fat and protein intake on liver fat in people at risk of unhealthy aging: 1-year results of a randomized controlled trial. Am J Clin Nutr. (2023) 117:785–93. doi: 10.1016/j.ajcnut.2023.01.010, 36804020

[78] Rinaldi R De Nucci S Donghia R Donvito R Cerabino N Di Chito M . Gender differences in liver steatosis and fibrosis in overweight and obese patients with metabolic dysfunction-associated steatotic liver disease before and after 8 weeks of very low-calorie ketogenic diet. Nutrients. (2024) 16:1408. doi: 10.3390/nu16101408, 38794646 PMC11123918

[79] Goldberg IJ Ibrahim N Bredefeld C Foo S Lim V Gutman D . Ketogenic diets, not for everyone. J Clin Lipidol. (2021) 15:61–7. doi: 10.1016/j.jacl.2020.10.005, 33191194 PMC7887024

[80] Fernández-Verdejo R Mey JT Ravussin E. Effects of ketone bodies on energy expenditure, substrate utilization, and energy intake in humans. J Lipid Res. (2023) 64:100442. doi: 10.1016/j.jlr.2023.100442, 37703994 PMC10570604

[81] Li X Yang J Zhou X Dai C Kong M Xie L . Ketogenic diet-induced bile acids protect against obesity through reduced calorie absorption. Nat Metab. (2024) 6:1397–414. doi: 10.1038/s42255-024-01072-1, 38937659

[82] Fuchs CD Trauner M. Role of bile acids and their receptors in gastrointestinal and hepatic pathophysiology. Nat Rev Gastroenterol Hepatol. (2022) 19:432–50. doi: 10.1038/s41575-021-00566-7, 35165436

[83] Alimov S Tursunov D Jurayev M. The effect of ketogenic diet on the hepatobiliary system: biochemical aspect. IMRAS. (2024) 7:123–5. doi: 10.5281/zenodo.14207408

[84] Andrewski E Cheng K Vanderpool C. Nutritional deficiencies in vegetarian, gluten-free, and ketogenic diets. Pediatr Rev. (2022) 43:61–70. doi: 10.1542/pir.2020-004275, 35102403

[85] Kiani AK Dhuli K Donato K Aquilanti B Velluti V Matera G . Main nutritional deficiencies. J Prev Med Hyg. (2022) 63:E93–E101. doi: 10.15167/2421-4248/jpmh2022.63.2S3.2752, 36479498 PMC9710417

[86] De Nucci S Bonfiglio C Donvito R Di Chito M Cerabino N Rinaldi R . Effects of an eight week very low-calorie ketogenic diet (VLCKD) on white blood cell and platelet counts in relation to metabolic dysfunction-associated steatotic liver disease (MASLD) in subjects with overweight and obesity. Nutrients. (2023) 15:4468. doi: 10.3390/nu15204468, 37892542 PMC10610501

[87] Sripongpun P Churuangsuk C Bunchorntavakul C. Current evidence concerning effects of ketogenic diet and intermittent fasting in patients with nonalcoholic fatty liver. J Clin Transl Hepatol. (2022) 10:730–9. doi: 10.14218/JCTH.2021.00494, 36062288 PMC9396320

[88] Georgiev A Chervenkov L Koleva D Anastasova V. Obesity control and liver health in breast cancer: normalized hepatic elasticity after ketogenic diet. Heliyon. (2023) 9:e20449. doi: 10.1016/j.heliyon.2023.e20449, 37780747 PMC10539953

