# Supplementary figures and images for: Mapping research landscapes: a bibliometric and visual analysis of ketogenic diet interventions in liver health (2013–2024)
Source: Front Nutr. 2025 Dec 23;12:1652271. doi: 10.3389/fnut.2025.1652271 (PMC12771114; doi:10.3389/fnut.2025.1652271)

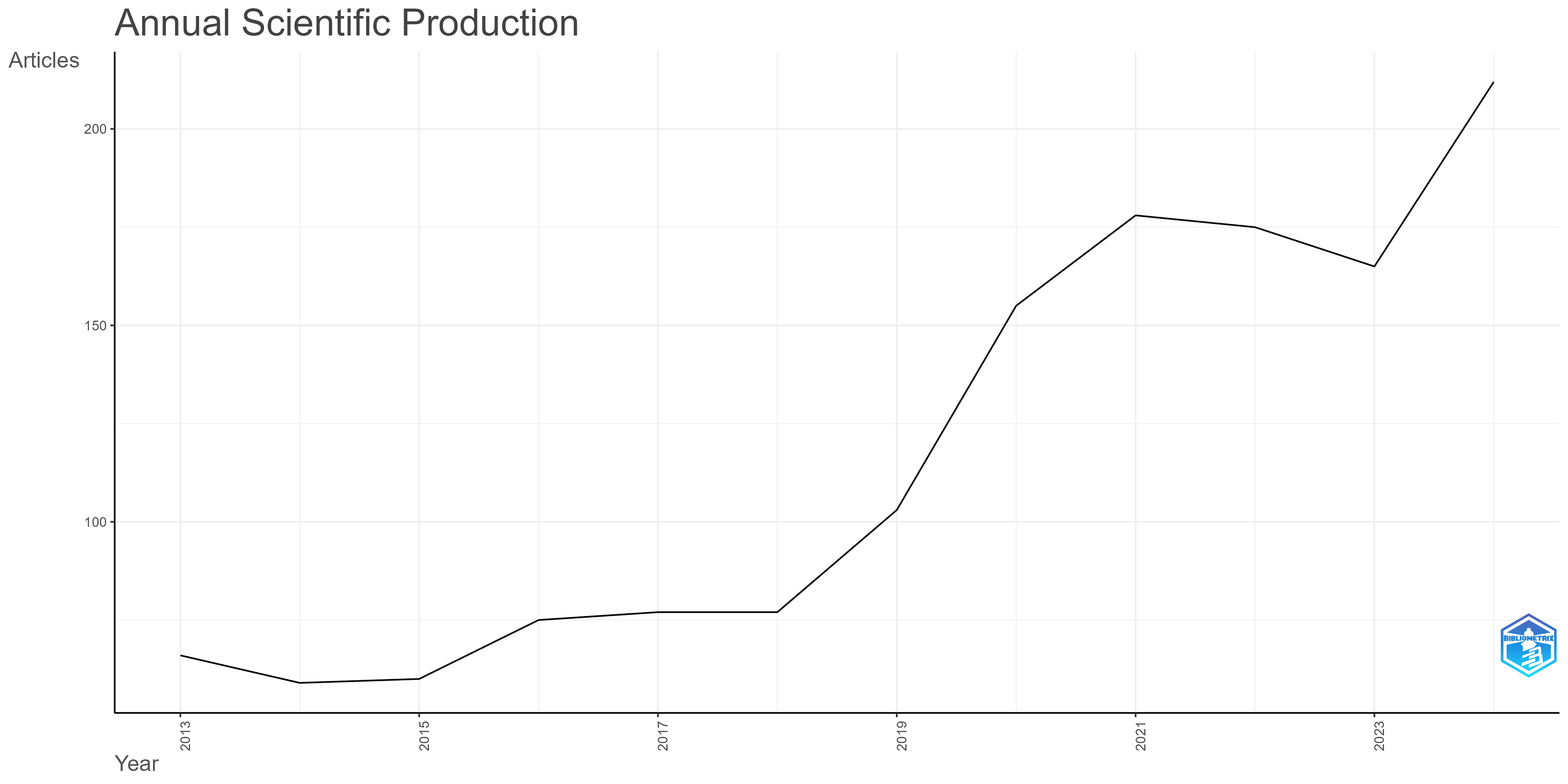

Supplement: Supplementary file 1 [file Image_1.TIF]

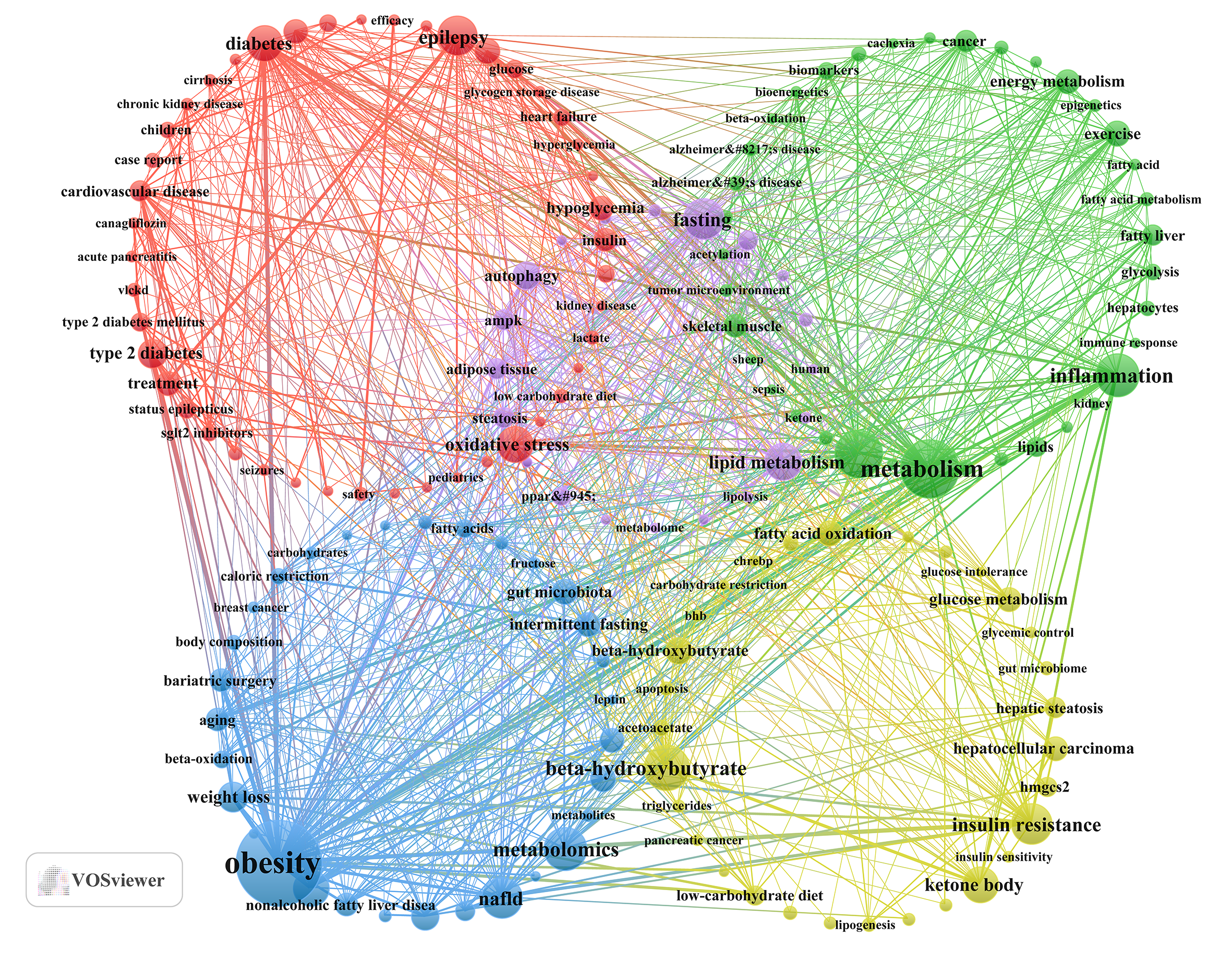

Supplement: Supplementary file 2 [file Image_2.TIF]
