# Supplementary material for: Mapping research landscapes: a bibliometric and visual analysis of ketogenic diet interventions in liver health (2013–2024)
Source: Front Nutr. 2025 Dec 23;12:1652271. doi: 10.3389/fnut.2025.1652271 (PMC12771114; doi:10.3389/fnut.2025.1652271)
Supplement: Supplementary file 3 [file Table_1.DOCX]

The research retrieval fomular of the field of TCM in PD (**WoSCC database**)

| Retrieve Expression | Results |
| --- | --- |
| ((((TS=("Ketogenic Diet*")) OR TS=(Ketogsis*)) OR TS="Ketone Body") | 5922 |
| (TS=(Liver*)) OR TS=(Hepar*) | 526445 |
| (((#1 AND #2) AND DOP=(2013-01-01/2024-07-21)) AND DT=(Article OR Review)) AND LA=(English) | 561 |

The research retrieval fomular of the field of TCM in PD (**Scopus database**)

( TITLE-ABS-KEY ( "Ketogenic Diet*" OR ketogsis* OR "Ketone Body" ) ) AND ( TITLE-ABS-KEY ( liver* OR hepar* ) ) AND PUBYEAR > 2012 AND PUBYEAR < 2025 AND ( LIMIT-TO ( DOCTYPE , "ar" ) OR LIMIT-TO ( DOCTYPE , "re" ) ) AND ( LIMIT-TO ( LANGUAGE , "English" ) )

Results: 1402
