# Supplementary material for: Mapping research landscapes: a bibliometric and visual analysis of ketogenic diet interventions in liver health (2013–2024)
Source: Front Nutr. 2025 Dec 23;12:1652271. doi: 10.3389/fnut.2025.1652271 (PMC12771114; doi:10.3389/fnut.2025.1652271)
Supplement: Supplementary file 4 [file Table_2.DOCX]

Vosviewer parameters (**WoSCC database**)

In co-authorship network analysis, we set the following parameters: minimum number of documents of a country ≥1; minimum number of documents of an organization ≥2; minimum number of documents of an author≥3. In the co-citation of source analysis, we set the following parameters: minimum number of citations of a source ≥2. Additionally, in the co-occurrence of keyword analysis, the parameters were set as follows: minimum number of occurrences of a keyword ≥3, and we excluded “ketogenic diet”, “ketogenic”, “diets”, “liver” “ketone body”, “ketone body metabolism” “ketones” and “ketosis” keywords and merge synonymous keywords.

Vosviewer parameters (**Scopus database**)

for co-occurrence keyword analysis, a keyword needed a minimum of 5 occurrences, excluding keywords terms used in the search query as well as their synonyms
